# Supplementary material for: Cryo-EM reveals ligand induced allostery underlying InsP3R channel gating
Source: Cell Res. 2018 Nov 23;28(12):1158–70. doi: 10.1038/s41422-018-0108-5 (PMC6274648; doi:10.1038/s41422-018-0108-5)
Supplement: Supplementary file 1 — Supplementary Figure S1 [file 41422_2018_108_MOESM1_ESM.pdf]

SUPPLEMENTARY FIGURES and TABLES

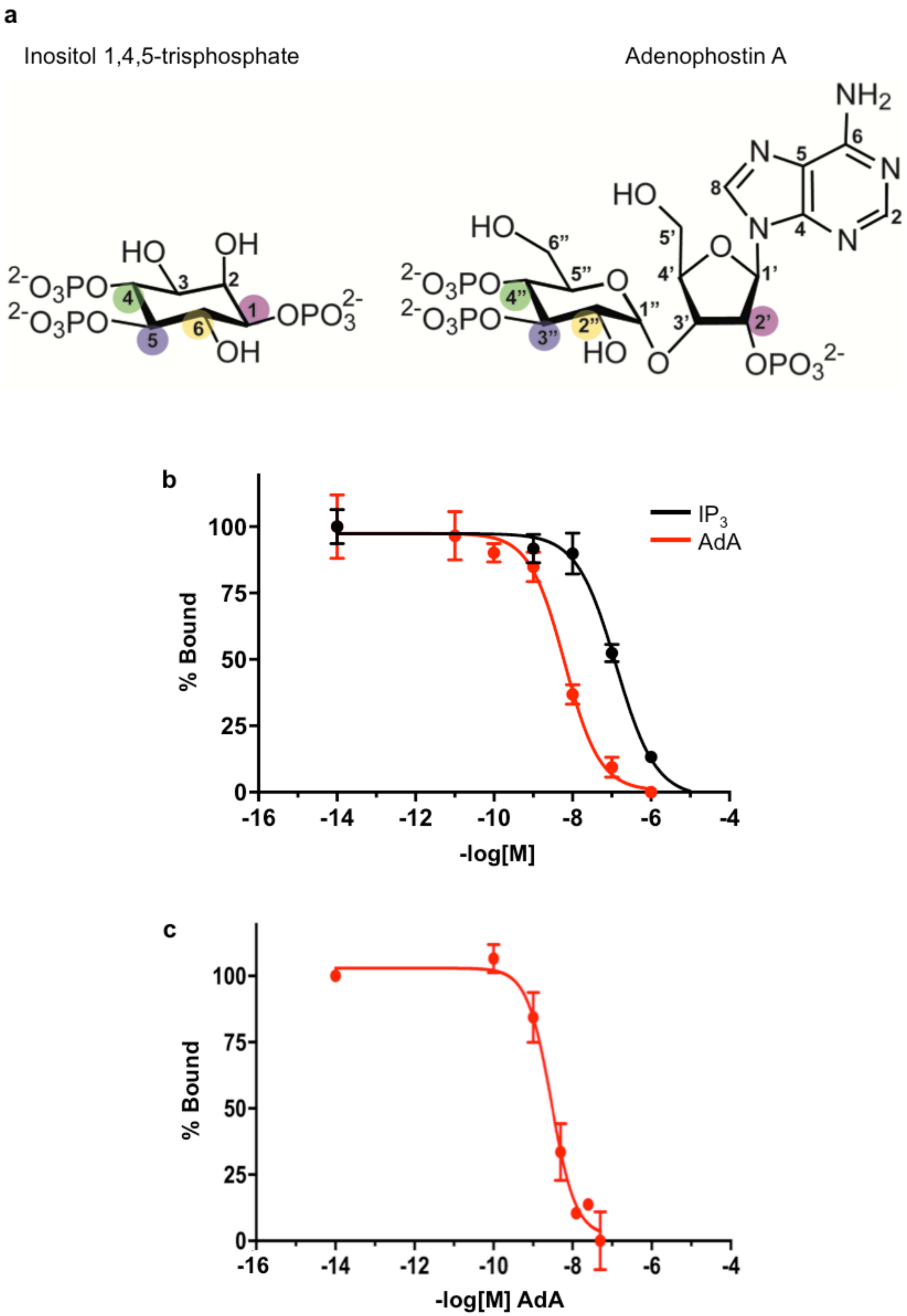

Supplementary information, Figure S1. Comparison of AdA and $InsP_3R1$. a Chemical structure of $InsP_3$ and AdA. Chemically equivalent moieties are coloured the same. Competitive ^3H-$InsP_3$ radioligand binding for rat cerebellar microsomal membranes (**b**) with IC_{50} for AdA = 7.9 ± 2 nM (n=3) and $InsP_3$= 127 ± 10 nM (n=3) and detergent solubilized, purified $InsP_3R1$ protein (**c**) with an IC_{50} for AdA = 7.5 ± 2 nM (n=3). Shown are data for one representative experiment for each condition.

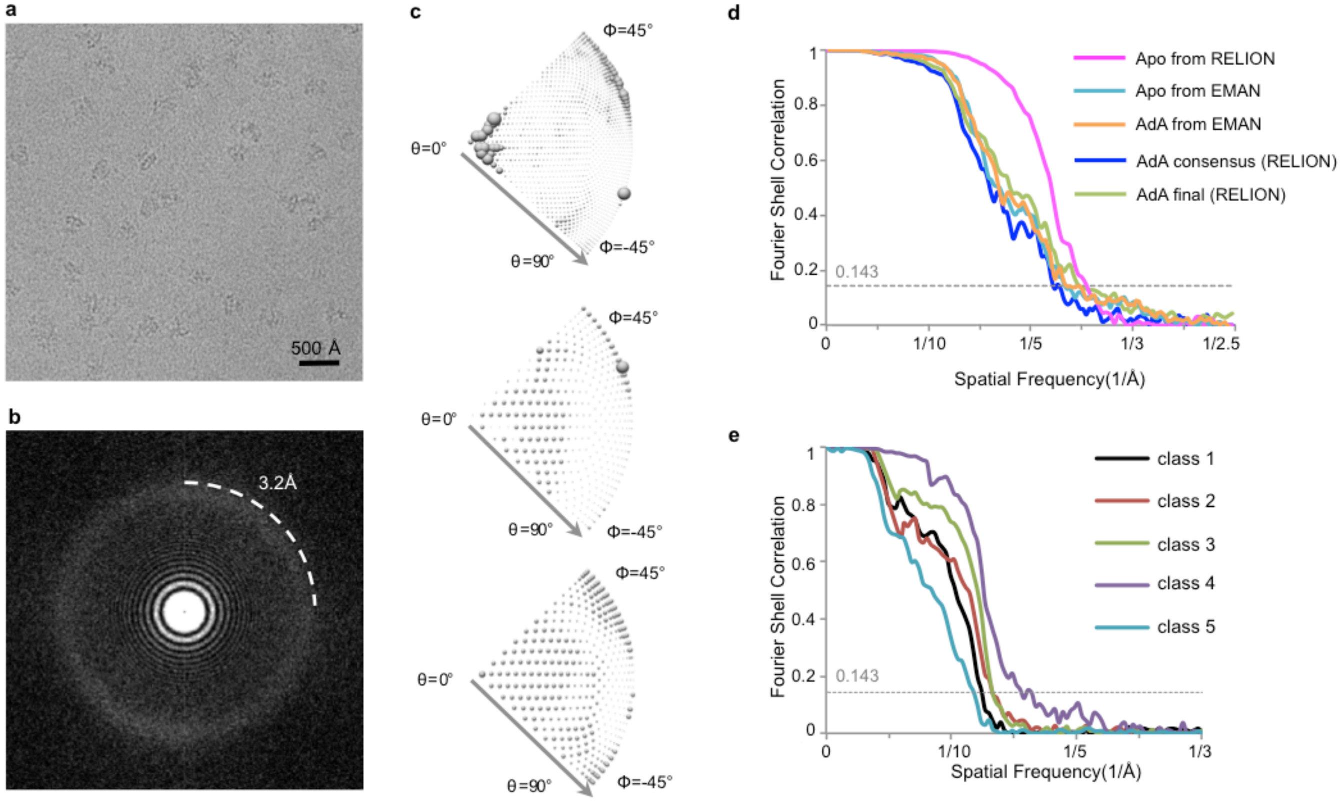

Supplementary information, Figure S2. Single-particle Cryo-EM analysis of Apo- and AdA-InsP$_3$R1. a, Representative 300 keV electron image of InsP$_3$R1 particles vitrified in the presence of activating ligands, AdA and Ca^{2+}. **b,** Fourier transform of image shown in (a). **c**, Euler angle distribution of particle orientations in the final refinement rounds in RELION. Each view is represented by a sphere, for which the size is proportional to the number of particles in a given orientation. Top panel: Apo-InsP$_3$R1 reconstruction; middle panel: consensus AdA-InsP$_3$R1 reconstruction; bottom panel: AdA-InsP$_3$R1 reconstruction after focused 3D classification (Material and Methods, Supplementary information, Figure S3). **d**, FSC curves for the cryo-EM 3D reconstructions. The resolution was estimated using the gold-standard FSC 0.143 criterion[50,51]. InsP$_3$R1 reconstructions from RELION: the Apo-map is magenta, 3.9 Å; the consensus AdA-map is dark blue, 4.5 Å; the final AdA-map is green, 4.1 Å. InsP$_3$R1 reconstructions from EMAN: the Apo-map is dark cyan, 4.3 Å; the AdA-map is orange, 4.2 Å. **e**, The gold-standard FSC curves for the cryo-EM maps generated without imposing c4 symmetry from the classes resulted from focused 3D classification (Materials and Methods; Supplementary information, Figure S3). **f**, The cryo-EM density maps of Apo-InsP$_3$R1 (upper panels) and AdA-InsP$_3$R1 (lower panels) are colour-coded based on ResMap (see Materials and Methods). The maps are viewed parallel to the membrane plane (left panels); the density slabs coincident with the 4-fold axis (indicated with dashed boxes in left panels) are shown in right panels.

f

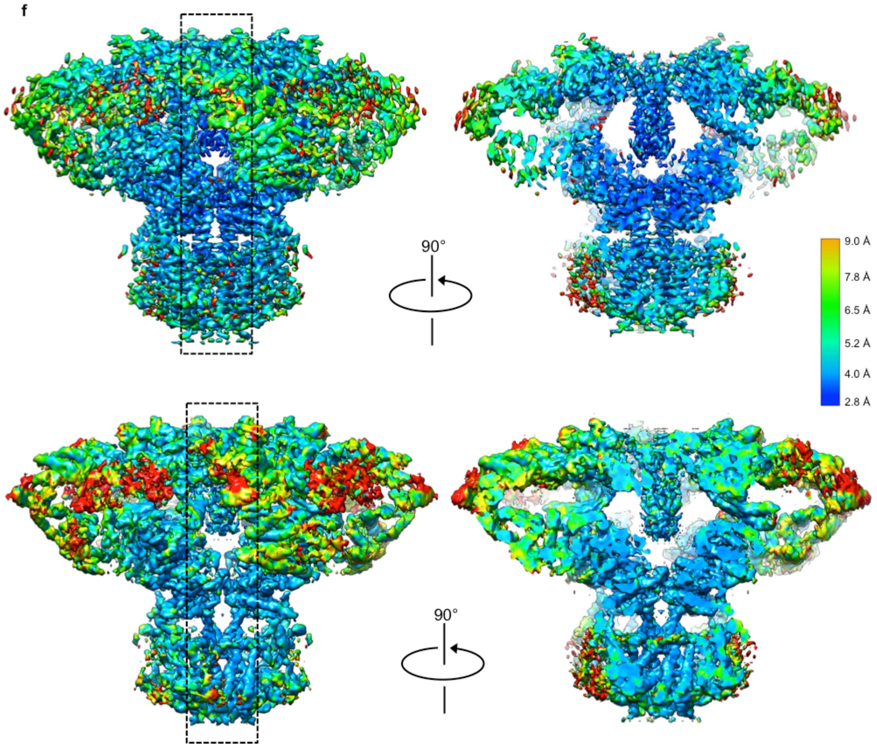

Supplementary information, Figure S2 (continued).

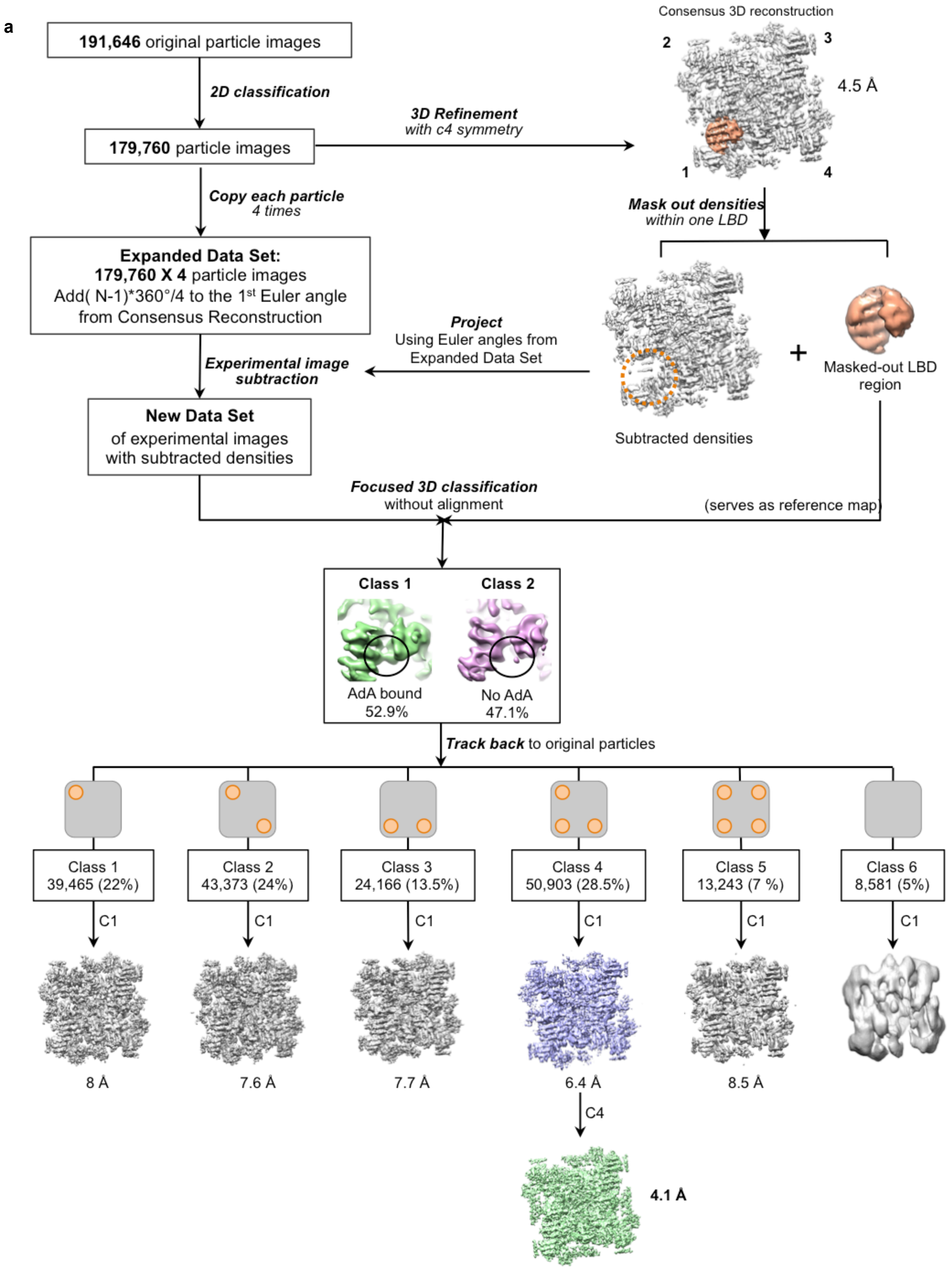

Supplementary information, Figure S3. 3D reconstruction of AdA-InsP$_3$R1. a, Workflow for 3D reconstruction of AdA-InsP$_3$R1. Signal-subtracted 3D classification was performed to resolve heterogeneity in the ligand-binding pocket (see Methods for details).

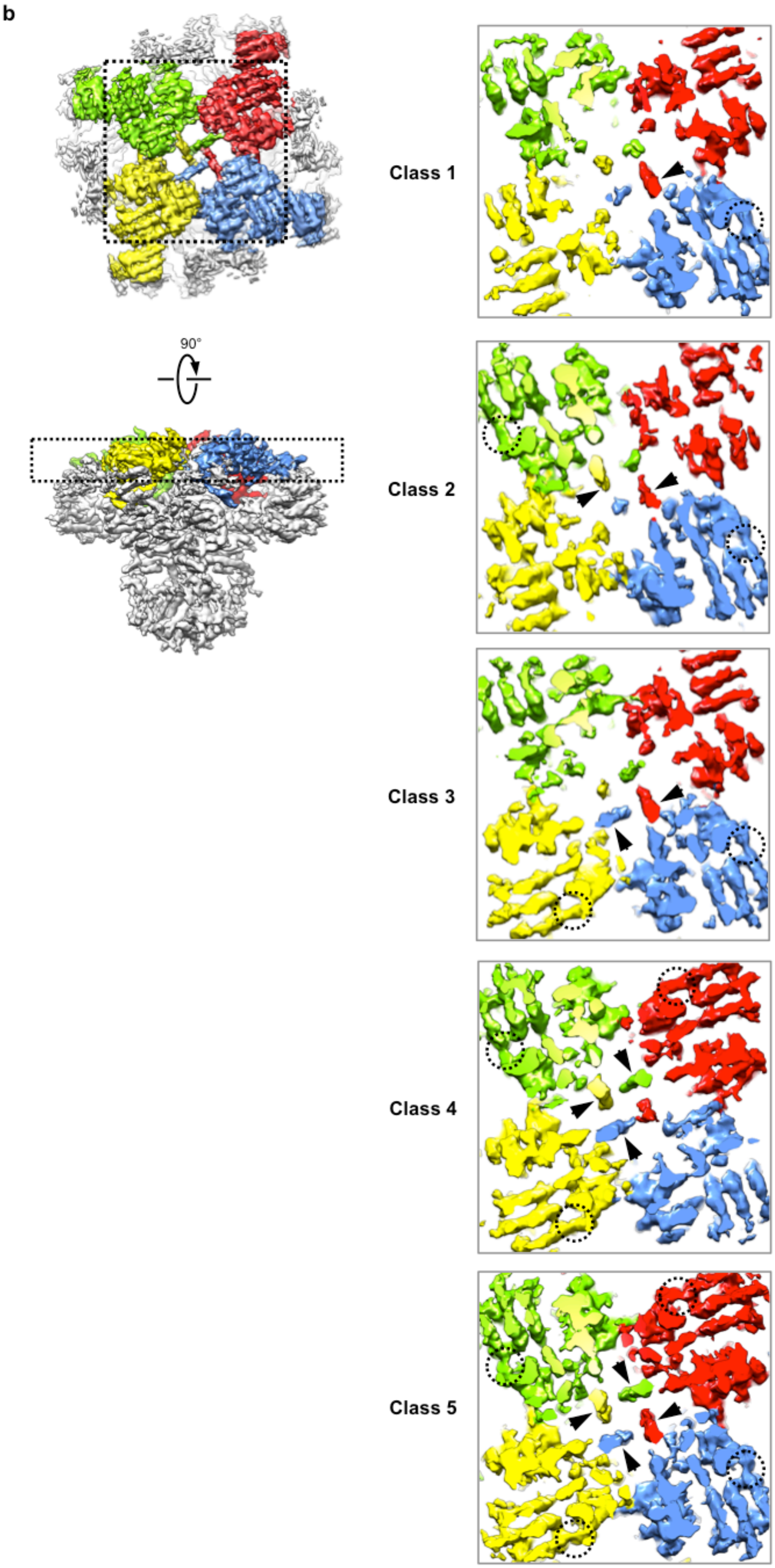

Supplementary information, Figure S3 (continued). b, Conformational changes at CTD/LBD interfaces visualized in 5 classes extracted via focused 3D classification as shown in 'a' (see Methods). Slices normal to the 4-four axis and viewed from cytosol are shown. Maps are colour-coded by subunits for the CTDs and LBDs as shown in the left panel. AdA-occupied LBDs are indicated with dashed line; arrowheads mark CTDs that are affected by ligand-binding. Noteworthy, ligand-binding causes structural rearrangement of the CTD of the neighboring subunit located counter-clockwise with respect to the AdA-occupied LBD.

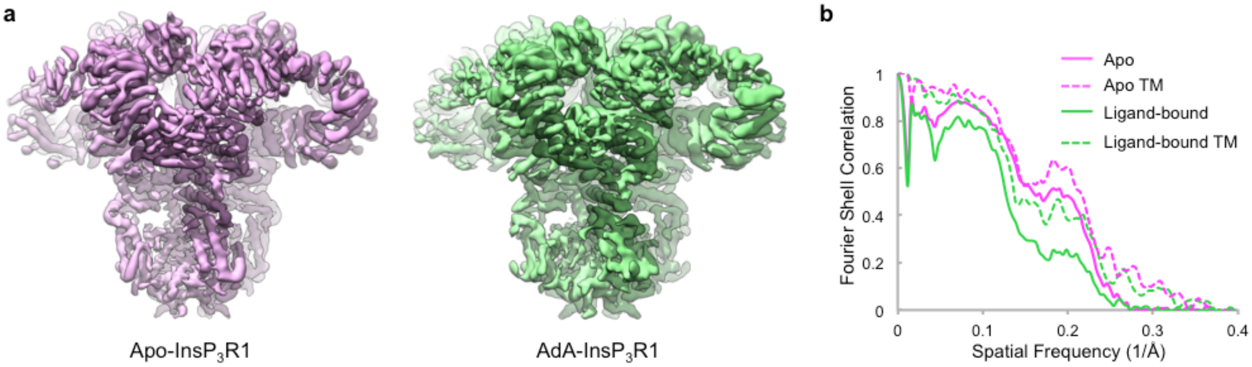

Supplementary information, Figure S4. Composite cryo-EM density maps of InsP$_3$R1. a, Isosurface rendering of the composite cryo-EM density maps for Apo- (left panel) and AdA-InsP$_3$R1 (right panel) (see Methods); the maps are viewed parallel to the membrane plane with the cytosolic regions facing up. **b**, The FSC plots obtained for the final models (solid lines) and for the TM domains (dashed lines) when compared to the corresponding composite cryo-EM maps.

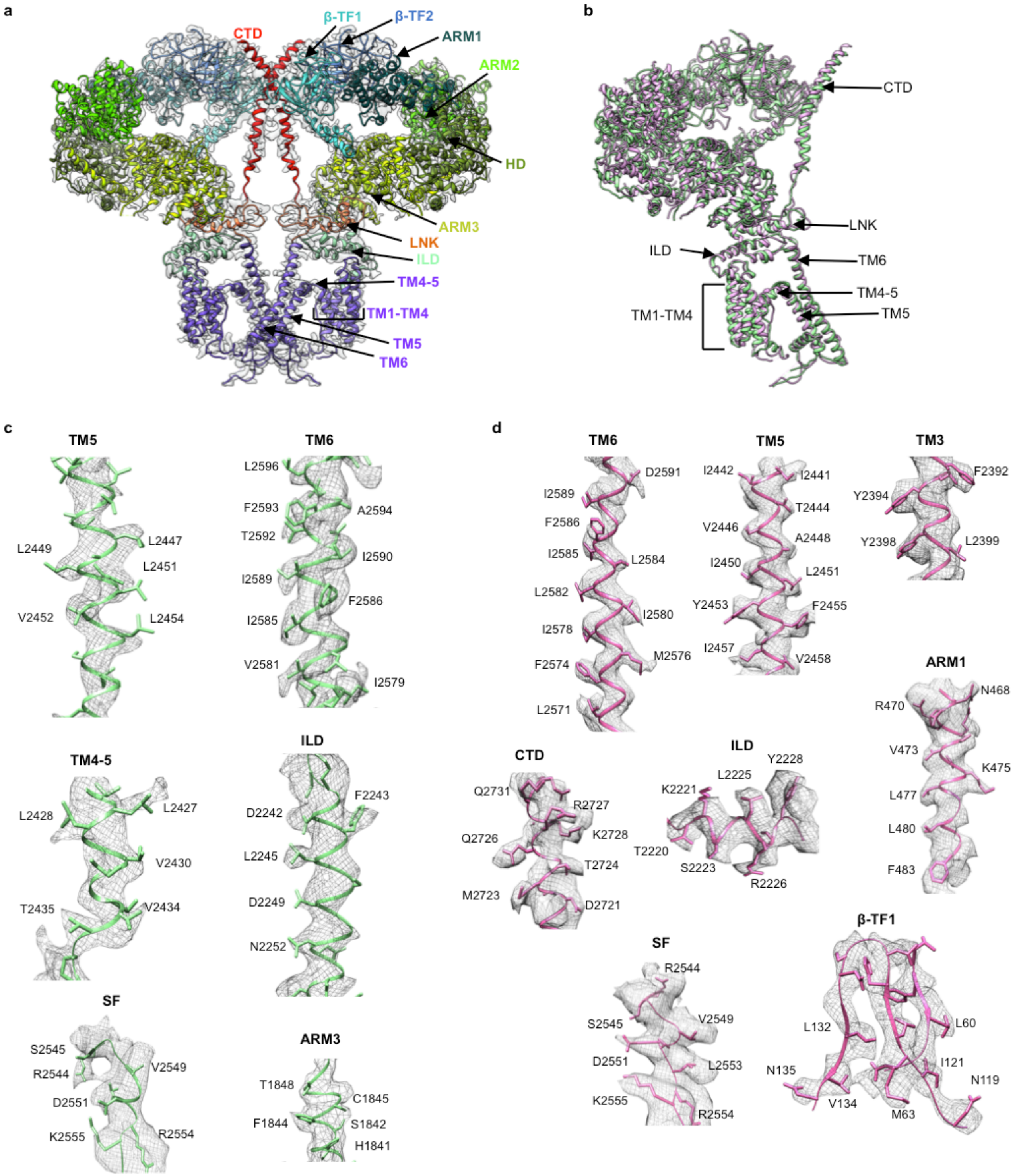

Supplementary information, Figure S5. Representative cryo-EM densities. a, The cryo-EM density map for AdA-InsP$_3$R1 is overlaid with the model; shown are two opposing subunits. The domains are colour-coded and labeled according to Supplementary information, Table S2. **b,** Models for one subunit of Apo- (light purple) and AdA-InsP$_3$R1 (green) are overlaid. **c-d**, Representative cryo-EM densities for selected regions are overlaid with corresponding models for AdA- (**c**) and Apo-InsP$_3$R1 (**d**).

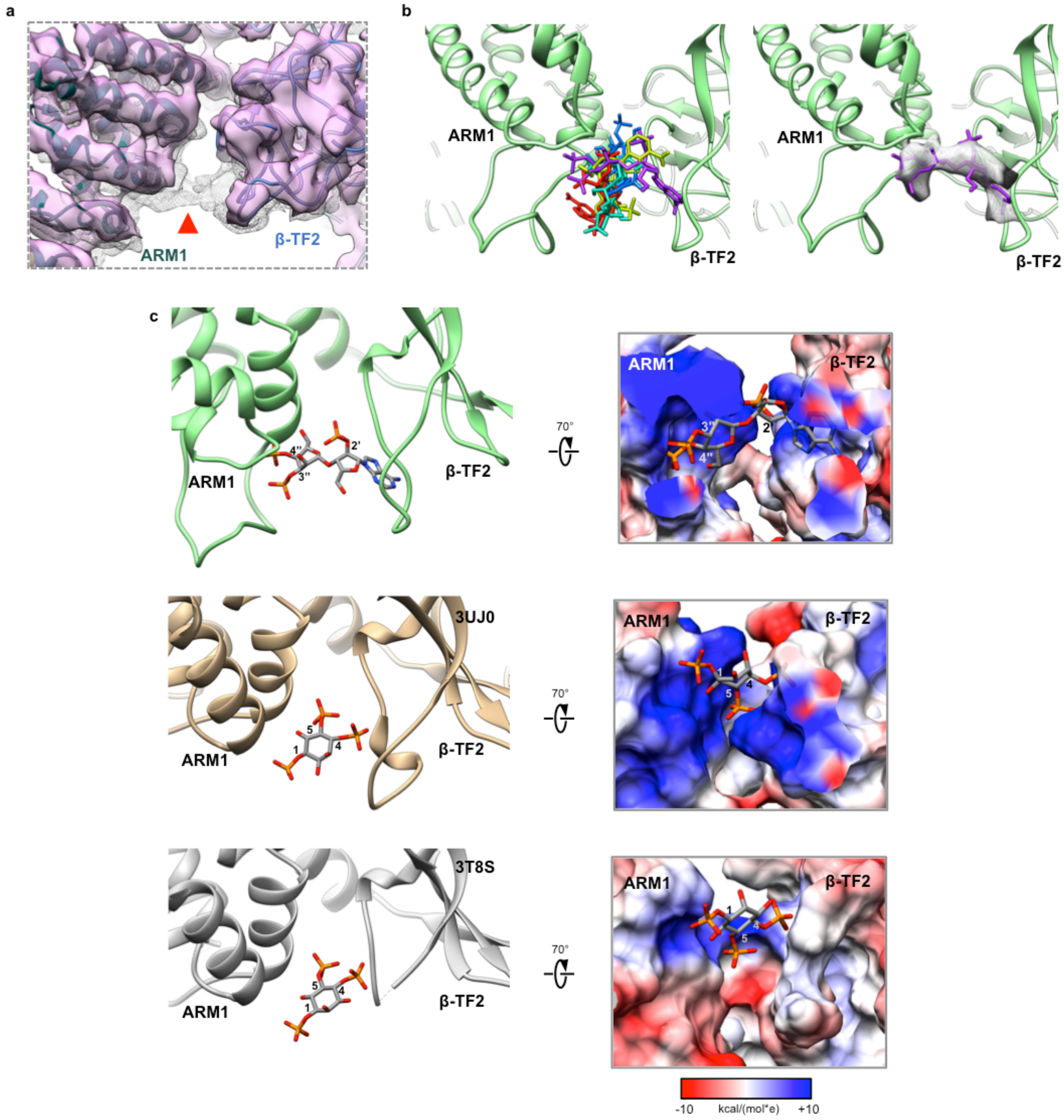

Supplementary information, Figure S6. Identification and characterization of the AdA binding pocket. a, Cryo-EM density maps for the ligand-binding pocket in the Apo- (purple) and AdA- (grey mesh) are overlaid; Apo-InsP$_3$R1 is depicted with ribbon model coloured by domain. The bridging density visualized between the ARM1 and β-TF2 domains in AdA-InsP$_3$R1 map is marked with red arrowhead. **b,** Several candidate positions for docking the AdA molecule generated using AutoDock Vina[71] (left panel) are displayed within the AdA-InsP$_3$R1 LBD structure (green). The right panel shows the final AdA molecular docking and its fit to the difference map density. **c**, Structures of isolated InsP$_3$-bound LBDs compared with the AdA-bound InsP$_3$R1 cryo-EM structure. Top panels: AdA-InsP$_3$R1 (green ribbon), middle panels: 3UJ0 (tan ribbon); bottom panels: 3T8S (grey ribbon). InsP$_3$ and AdA are colour-coded by element: (phosphorous - orange; oxygen - red; nitrogen - blue; carbon - grey; phosphates are labeled as indicated in Supplementary information Figure S1a). The right panels show the surface electrostatic charges calculated for the corresponding ligand binding pockets.

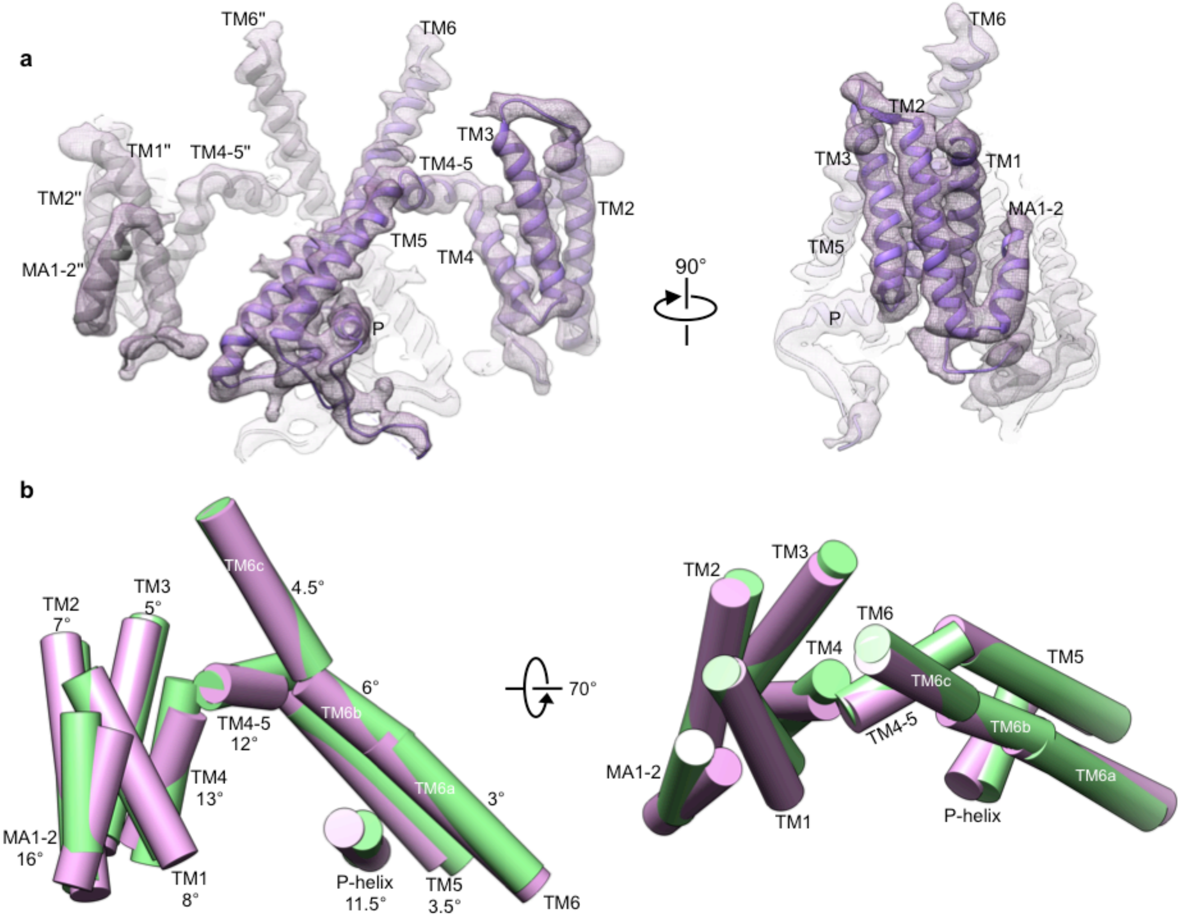

Supplementary information, Figure S7. Cryo-EM density map for the TM region of Apo-InsP$_3$R1. a, Two views of the Apo-InsP$_3$R1 cryo-EM density map for the TM1-TM6 helices and interconnecting loops of two opposing subunits; viewed parallel to the membrane plane with the lumenal side facing down. **b**, Superimposition of TM helices from one subunit of Apo (light purple) and AdA-bound (green) structures; the helices are depicted as cylinders in two views: parallel to the membrane plane (left, cytosolic side up), and rotated 70° (right, viewed from the cytosol). Changes in a rigid body tilt for each TM helix in AdA-InsP$_3$R1 with respect to the orientation in the Apo-state are indicated. TM6 helix exhibits three different tilt angles defined for TM6a (E2560-I2578), TM6b (I2579-I2590), TM6c (D2691-K2608).

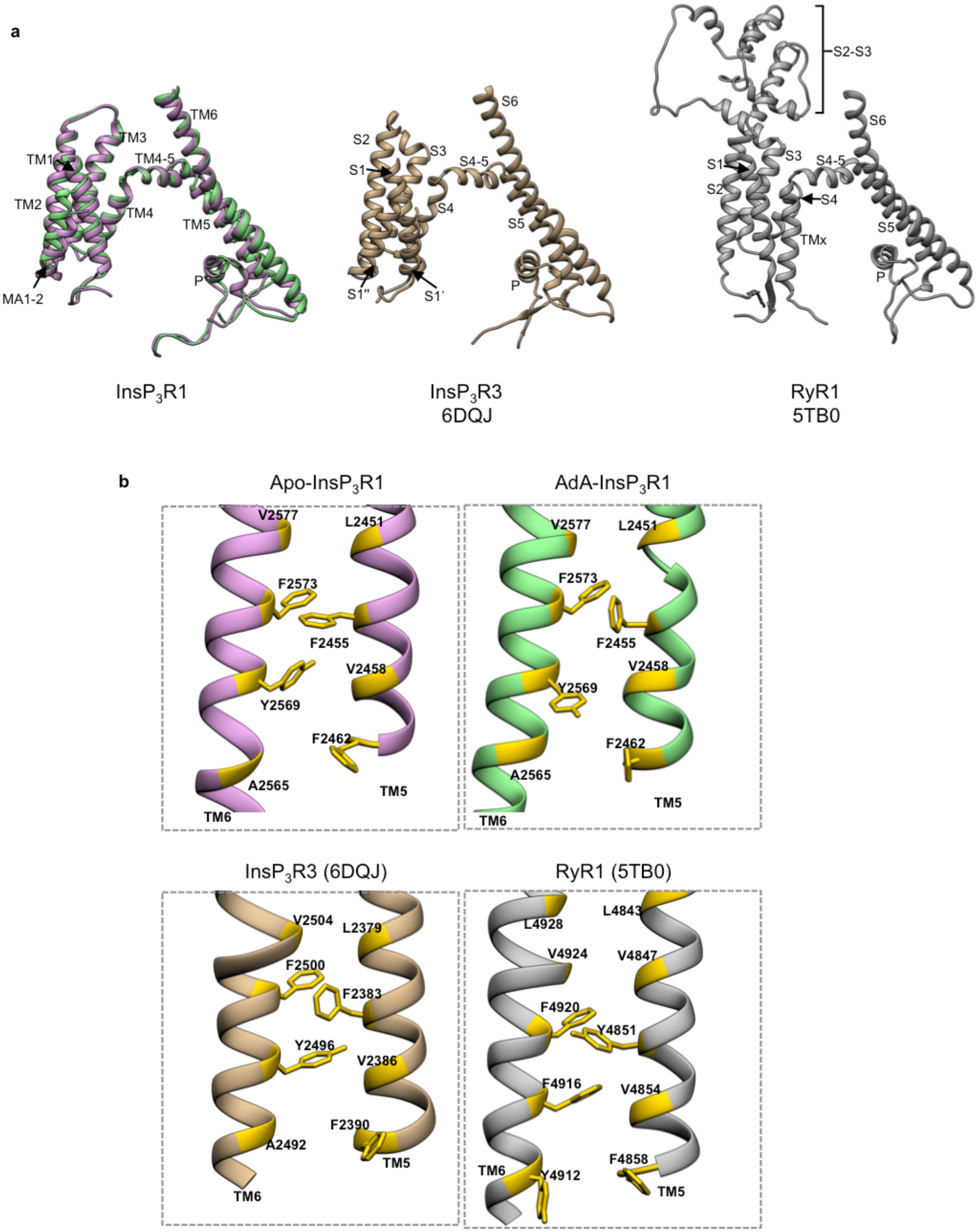

Supplementary information, Figure S8. Comparative analysis of the TM domains. a, Six TM domain structure in InsP$_3$R1 (apo-structure – light purple, AdA-structure – green), InsP$_3$R3 (6DQJ) and RyR1 (5TB0). **b,** TM6 and TM5 helices from the same subunit exhibit hydrophobic interactions and aromatic side-chain stacking in the lumenal leaflet: Apo-InsP$_3$R1 (light purple), AdA- InsP$_3$R1 (green); InsP$_3$R3 (tan), RyR1 (grey). Hydrophobic residues within the interface are colored yellow and aromatic side-chains are shown.

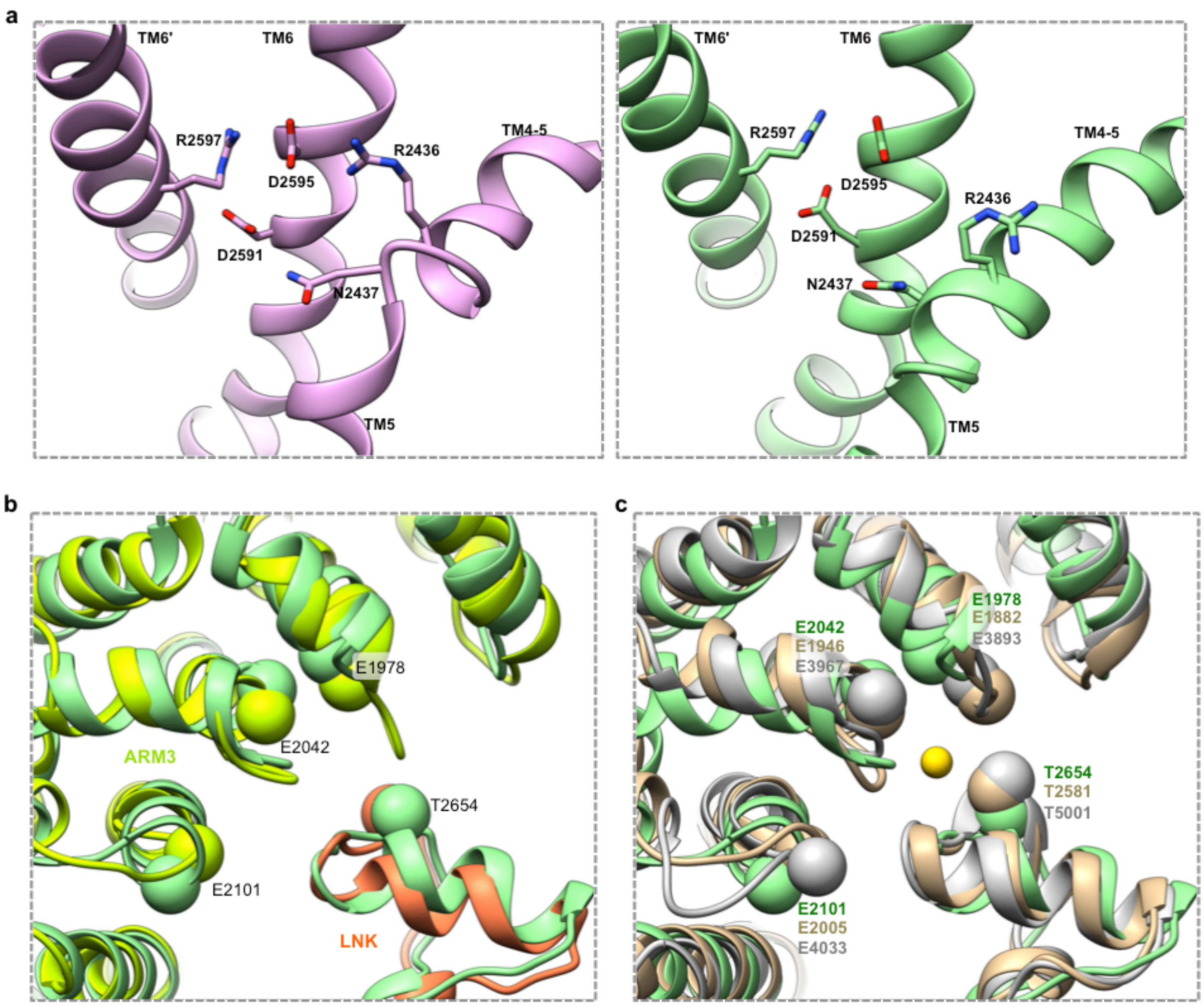

Supplementary information, Figure S9. Inter- and intra-subunit contacts within the pore region. a, Residues on the neighboring TM6 helix within 5 Å of the R2597 of TM6' are shown with side-chains. The lateral membrane-associated TM4-5 is located in close proximity to the TM6-TM6' interaction site, whereby each TM4-5 helix is positioned to interact with the TM6 helix from the same subunit. Apo and AdA-bound $InsP_3R1$ structures are depicted as light purple (left) and green (right), respectively. Conformational changes observed within this region upon ligand binding may serve to communicate with the gate. **b**, Zoomed-in view of the putative Ca^{2+} sensor region in ARM3-LNK domains of AdA-$InsP_3R1$ (green) that is superimposed with the same domain in the Apo-$InsP_3R1$ (colour-coded by domains). Residues that may play a role in Ca^{2+} binding based on a structure-based sequence alignment with RyR1[6] are labeled and their Cα atoms depicted as spheres. **c,** Structural alignment of the putative Ca^{2+} sensor regions for AdA-$InsP_3R1$ (green), RyR1 (5T15, grey) and $InsP_3R3$ (6DR2, tan). Residues involved in Ca^{2+} coordination in RyR1 and $InsP_3R3$ and the corresponding conserved residues in $InsP_3R1$ are indicated as spheres and labeled in the same color as the model. The Ca^{2+} ion modeled in RyR1 and $InsP_3R3$ structures is depicted as yellow sphere.

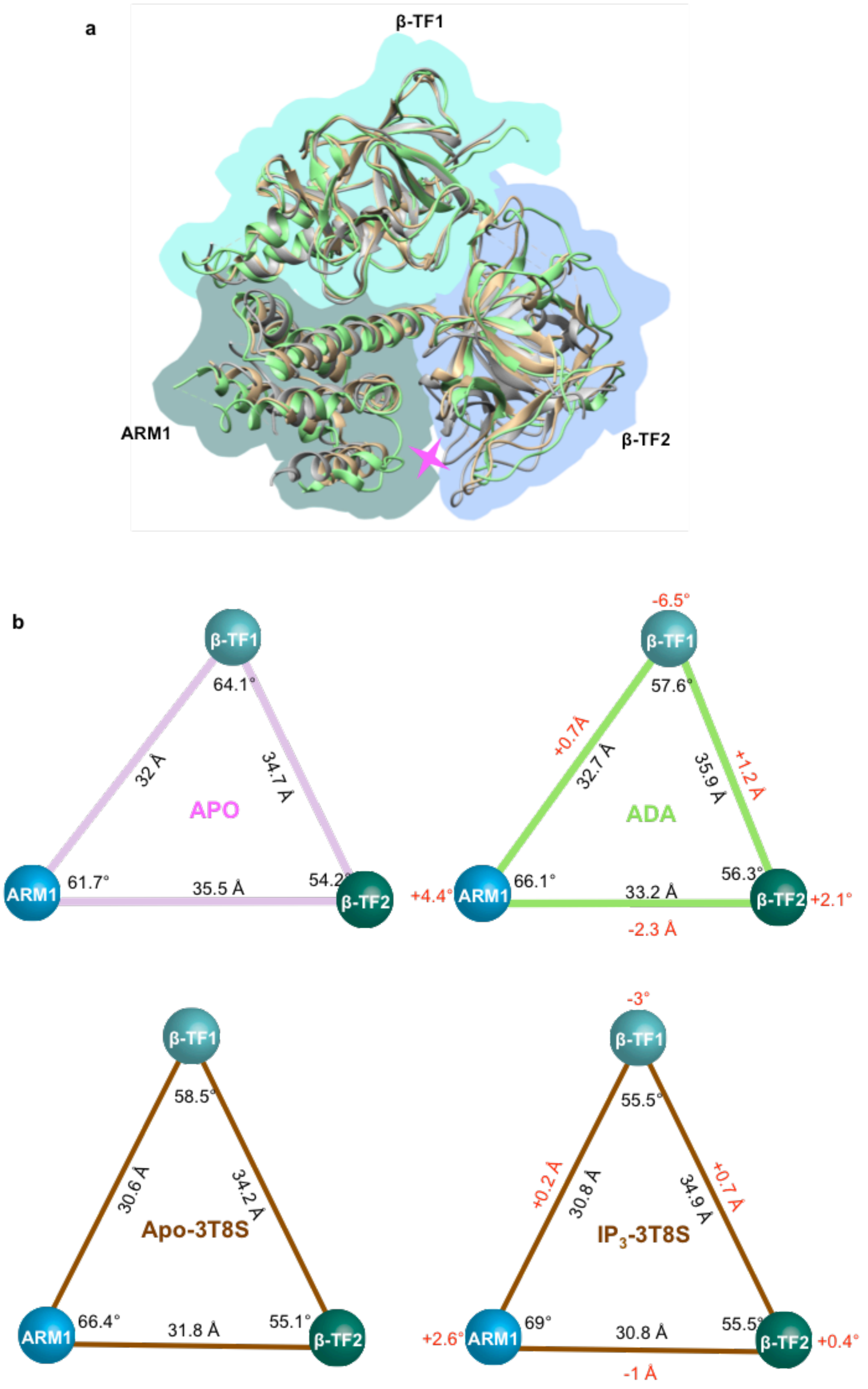

Supplementary information, Figure S10. Comparative analysis of AdA- and InsP$_3$- bound ligand binding domains. a, The triangular arrangement of ligand-binding domains within tetrameric InsP$_3$R structure: AdA-InsP$_3$R LBD (green), 3UJ0 (grey) and 3T8S (tan). **b,** Angular relationships and relative distances between β-TF1, ARM1 and β-TF2 domains in Apo- and AdA-bound structures of InsP$_3$R1 (upper panels) and in the Apo- and InsP$_3$-bound crystal structure of isolated LBD (3T8S; lower panels) as estimated based on the center of mass for each domain. It is noticeable, that AdA ligand binding results in a greater domain closure between ARM1 and β-TF2 than InsP$_3$. This results in a decrease in the angular relationship of ARM1/β-TF1/β-TF2 domains while the β-TF1/β-TF2/ARM1 angle increases.

Supplementary Information, Table S1.
Cryo-EM structure determination and model statistics

Data collection	Apo	Ligand-bound
Microscope	FEI Polara	FEI Polara
Voltage (kV)	300	300
Detector	Gatan K2 Summit	Gatan K2 Summit
Magnification	31,000	31,000
Pixel size (Å)	1.26 (0.63)	1.26 (0.63)
Total dose (e/Å^2)	38	44
Dose rate (electrons/pixel/sec)	~10	~10
Exposure time (sec)	6	7
Defocus range (μm)	-0.8 to -3.5	-0.9 to -3.2
Movie stacks	9,823	14,686
Subframes	30	35
**Data Processing**	**Apo**	**Ligand-bound**
Defocus determining software	CTFFIND3	CTFFIND3
Motion correction software	dosefgpu_driftcorr	dosefgpu_driftcorr
Refine software	RELION 1.4	RELION 1.4
Particle picking software	e2boxer.py	e2boxer.py
Number of boxed particles	207,914	191,646
Number of particles after 2D classification	144,194	179,760
Number of particles in final reconstruction	65,438	179,760
Symmetry imposed	C4	C4
Map resolution (Å)	3.9	4.5
**EMAN Refinement**	**Apo**	**Ligand-bound**
Number of particles:		
initial refinement	144,194	179,760
final reconstruction	100,615	38,405
Symmetry imposed	C4	C4
Map resolution (Å)	4.3	4.2
**3D Refinement**		**Class 4**
Refine software		RELION 1.4
Number of particles in final reconstruction		50,903
Symmetry imposed		C1
Map resolution (Å)		6.4
Symmetry imposed		C4
Map resolution (Å)		4.1
**Atomic Model**	**Apo**	**Ligand-bound**
Ramachandran outliers	0.34%	1.13%
Ramachandran favored	84.89%	84.89%
Rotamer outliers	1.49%	3.45%
C-beta deviations	7	32
RMS(bonds)	0.01	0.007
RMS(angles)	1.62	1.64
Molprobity score	2.36	3.10
Molprobity clashscore	11.27	35.36

Supplementary information, Table S1. Summary of Cryo-EM data collection, image processing, 3D reconstruction and model statistics.

Supplementary Information, Table S2.

Domain abbreviation	Domain Name	Spanning Residues
β-TF1	beta trefoil domain 1	M1-K225
β-TF2	beta trefoil domain 2	W226-V435
ARM1	armadillo repeat 1	S436-L665
HD	helical domain	I707-S1008
ARM2	armadillo repeat 2	P1025-R1538
ARM3	armadillo repeat 3	R1598-H2192
ILD	intervening lateral domain	T2193-V2265
TMD	transmembrane domains	L2266-K2608
LNK	linker domain	T2609-M2681
CTD	C-terminal domain	S2682-A2750

Supplementary Table S2. Definition of domains in $InsP_3R1$ structure.

Supplementary Information, Table S3.

	This study	Hite *et al.*, 2018	Lin *et al.*, 2011	Seo *et al.*, 2012
**Method**	Cryo-EM	Cryo-EM	X-ray crystallography	X-ray crystallography
**Channel**	Rat InsP$_3$R1	Human InsP$_3$R3	Rat InsP$_3$R1	Rat InsP$_3$R1
**PDB**		6DQN	3T8S	3UJ0
**Ligand**	AdA	InsP$_3$	InsP$_3$	InsP$_3$
**β-TF2**<br>Loop	**Adenine**<br>T267-T276<br><br>**2' PO$_3^{2-}$**<br>R265	**4-PO$_3^{2-}$**<br>R266<br>T268<br>R270	**4-PO$_3^{2-}$**<br>R265<br>T267	**4-PO$_3^{2-}$**<br>R265<br>T267<br>R269
**ARM1**<br>Helix	**4'' PO$_3^{2-}$**<br>R504<br>Q507<br>K508<br><br>**2' PO$_3^{2-}$**<br>R511<br>K508	**5 PO$_3^{2-}$**<br>K507<br>R510<br><br>**1 PO$_3^{2-}$**<br>R503	**5 PO$_3^{2-}$**<br>R511	**5 PO$_3^{2-}$**<br>R504<br>R511<br><br>**1 PO$_3^{2-}$**<br>R504
**ARM1**<br>Loop	**3'' PO$_3^{2-}$**<br>D566<br>Y567<br>R568<br>K569<br><br>**4'' PO$_3^{2-}$**<br>Y567<br>N570<br>Q571	**1 PO$_3^{2-}$**<br>R568<br><br>**5 PO$_3^{2-}$**<br>Y567<br>K569	**1 PO$_3^{2-}$**<br>R568<br><br>**5 PO$_3^{2-}$**<br>Y567	**1 PO$_3^{2-}$**<br>R568<br><br>**5 PO$_3^{2-}$**<br>Y567<br>K569

Supplementary Table S3. Comparative analysis of InsP$_3$ and AdA interactions with LBD in X-ray crystallographic structures[18,19] and in cryo-EM structures of InsP$_3$R1 and InsP$_3$R3[20]. The table includes residues located within 5 Å from AdA molecule in the AdA-InsP$_3$R1 structure and may potentially coordinate the ligand in the binding pocket. Residues for coordination of InsP$_3$ were extracted from the publications indicated in the table.
