# Supplementary material for: Cryo-EM reveals ligand induced allostery underlying InsP3R channel gating
Source: Cell Res. 2018 Nov 23;28(12):1158–70. doi: 10.1038/s41422-018-0108-5 (PMC6274648; doi:10.1038/s41422-018-0108-5)
Supplement: Supplementary file 2 — Supplementary Figure S2 [file 41422_2018_108_MOESM2_ESM.pdf]

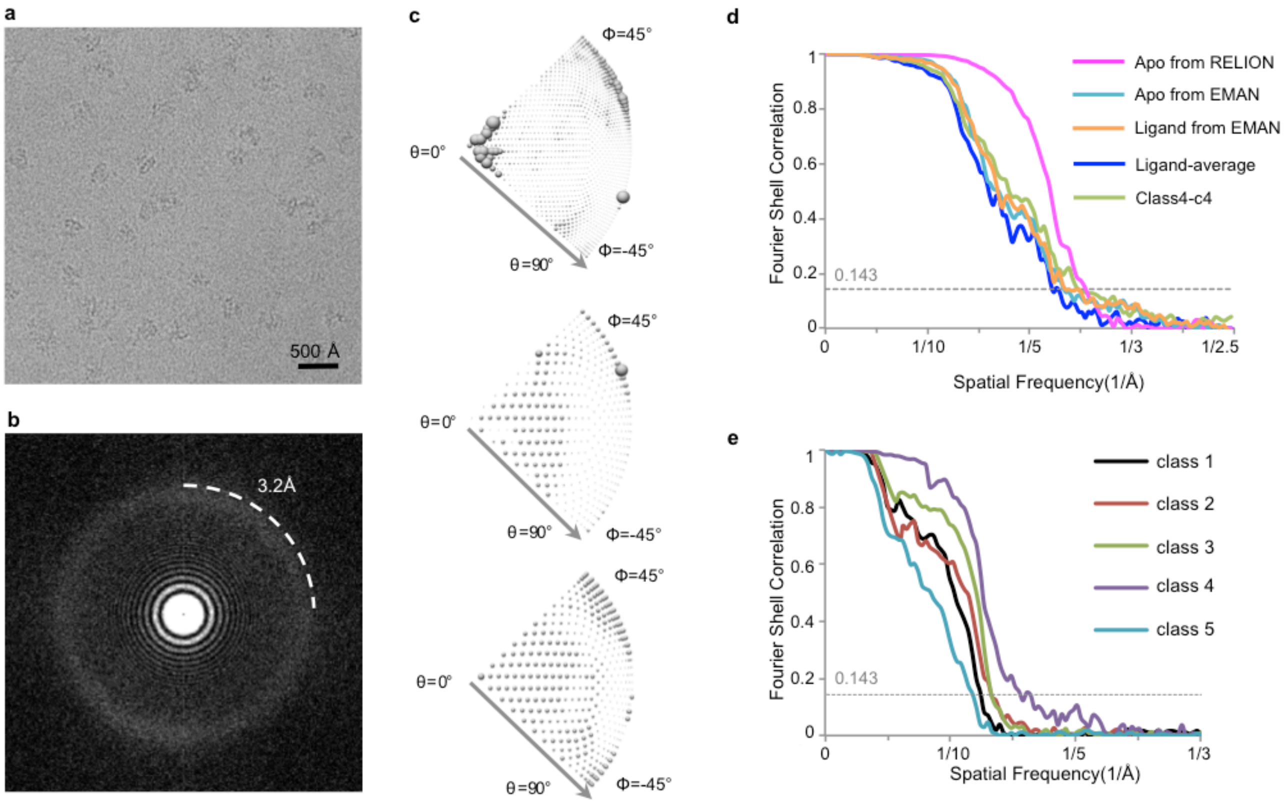

Supplementary information, Figure S2. Single-particle Cryo-EM analysis of Apo- and AdA-InsP_3R1. a, Representative 300 keV electron image of InsP_3R1 particles vitrified in the presence of activating ligands, AdA and Ca^{2+}. **b,** Fourier transform of image shown in (a). **c**, Euler angle distribution of particle orientations in the final refinement rounds in RELION. Each view is represented by a sphere, for which the size is proportional to the number of particles in a given orientation. Top panel: Apo-InsP_3R1 reconstruction; middle panel: consensus AdA-InsP_3R1 reconstruction; bottom panel: AdA-InsP_3R1 reconstruction after focused 3D classification (Material and Methods, Supplementary information, Figure S3). **d**, FSC curves for the cryo-EM 3D reconstructions. The resolution was estimated using the gold-standard FSC 0.143 criterion[50,51]. **e**, The gold-standard FSC curves for the cryo-EM maps generated without imposing c4 symmetry from the classes resulted from focused 3D classification (Materials and Methods; Supplementary information, Figure S3). **f**, The cryo-EM density maps of Apo-InsP_3R1 (upper panels) and AdA-InsP_3R1 (lower panels) are colour-coded based on ResMap (see Materials and Methods). The maps are viewed parallel to the membrane plane (left panels); the density slabs coincident with the 4-fold axis (indicated with dashed boxes in left panels) are shown in right panels.

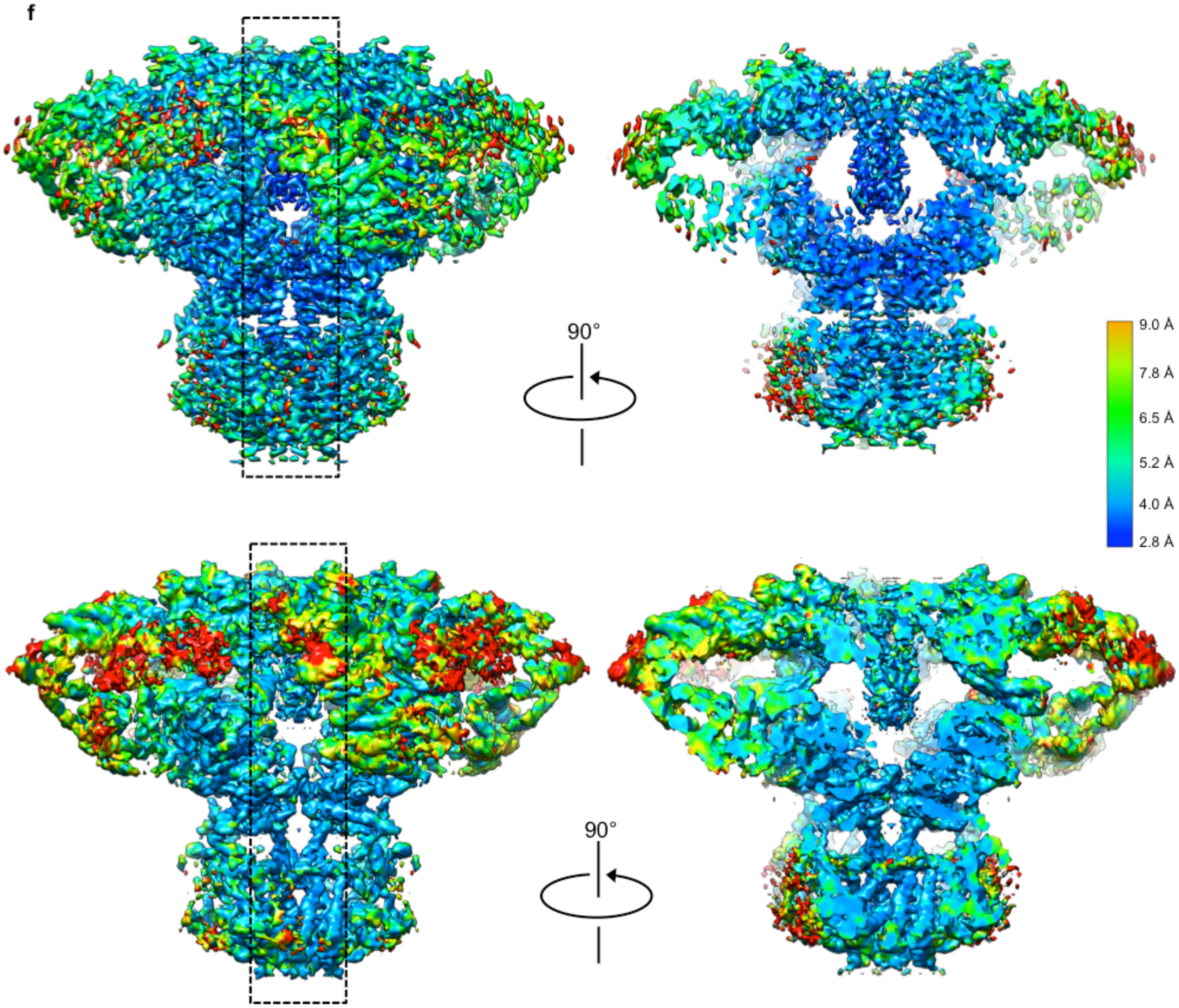

Supplementary information, Figure S2 (continued).
