# Supplementary material for: Cryo-EM reveals ligand induced allostery underlying InsP3R channel gating
Source: Cell Res. 2018 Nov 23;28(12):1158–70. doi: 10.1038/s41422-018-0108-5 (PMC6274648; doi:10.1038/s41422-018-0108-5)
Supplement: Supplementary file 3 — Supplementary Figure S3 [file 41422_2018_108_MOESM3_ESM.pdf]

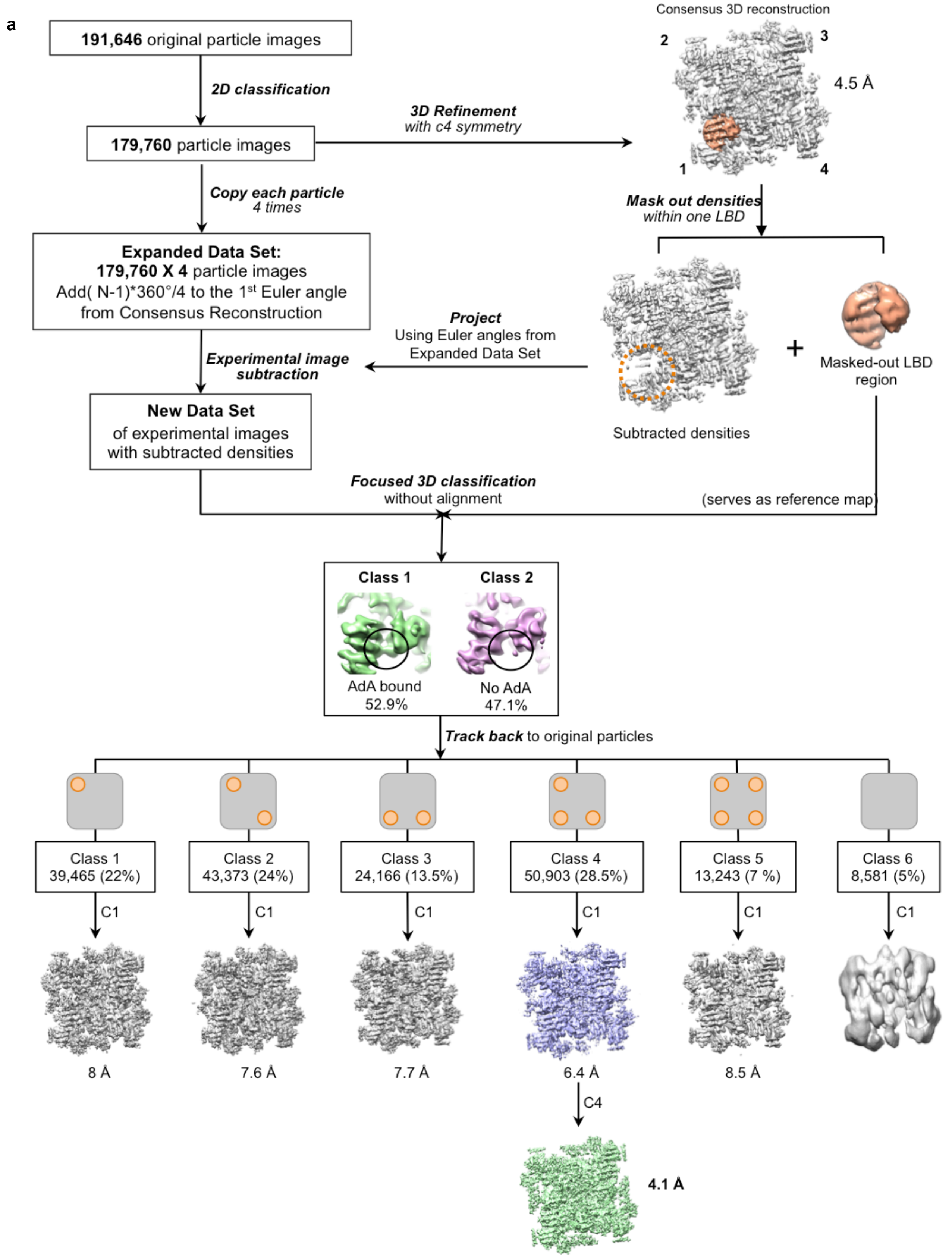

Supplementary information, Figure S3. 3D reconstruction of AdA-InsP$_3$R1. a, Workflow for 3D reconstruction of AdA-InsP$_3$R1. Signal-subtracted 3D classification was performed to resolve heterogeneity in the ligand-binding pocket (see Methods for details).

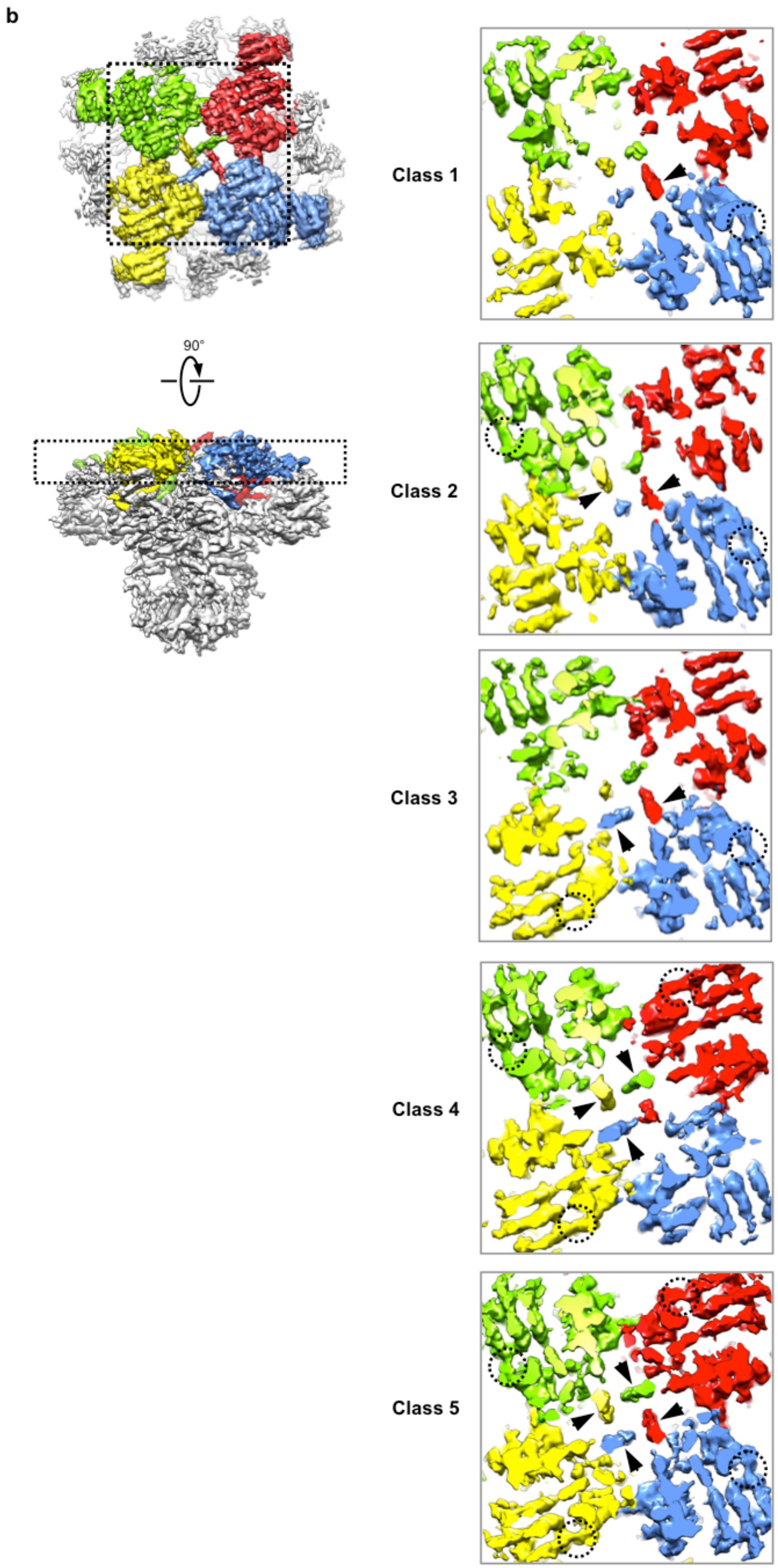

Supplementary information, Figure S3 (continued). b, Conformational changes at CTD/LBD interfaces visualized in 5 classes extracted via focused 3D classification as shown in 'a' (see Methods). Slices normal to the 4-four axis and viewed from cytosol are shown. Maps are colour-coded by subunits for the CTDs and LBDs as shown in the left panel. AdA-occupied LBDs are indicated with dashed line; arrowheads mark CTDs that are affected by ligand-binding. Noteworthy, ligand-binding causes structural rearrangement of the CTD of the neighboring subunit located counter-clockwise with respect to the AdA-occupied LBD.
