# Supplementary material for: Cryo-EM reveals ligand induced allostery underlying InsP3R channel gating
Source: Cell Res. 2018 Nov 23;28(12):1158–70. doi: 10.1038/s41422-018-0108-5 (PMC6274648; doi:10.1038/s41422-018-0108-5)
Supplement: Supplementary file 4 — Supplementary Figure S4 [file 41422_2018_108_MOESM4_ESM.pdf]

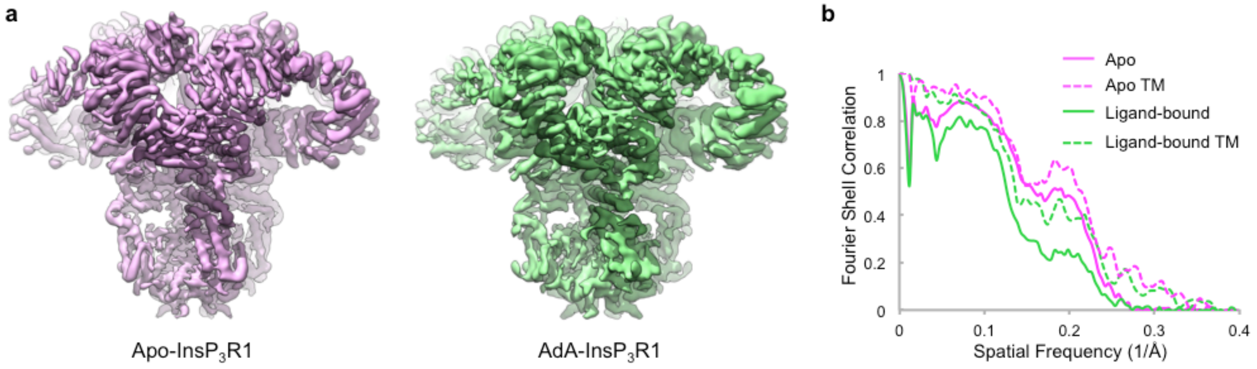

Supplementary information, Figure S4. Composite cryo-EM density maps of InsP$_3$R1. a, Isosurface rendering of the composite cryo-EM density maps for Apo- (left panel) and AdA-InsP$_3$R1 (right panel) (see Methods); the maps are viewed parallel to the membrane plane with the cytosolic regions facing up. **b**, The FSC plots obtained for the final models (solid lines) and for the TM domains (dashed lines) when compared to the corresponding composite cryo-EM maps.
