# Supplementary material for: Cryo-EM reveals ligand induced allostery underlying InsP3R channel gating
Source: Cell Res. 2018 Nov 23;28(12):1158–70. doi: 10.1038/s41422-018-0108-5 (PMC6274648; doi:10.1038/s41422-018-0108-5)
Supplement: Supplementary file 5 — Supplementary Figure S5 [file 41422_2018_108_MOESM5_ESM.pdf]

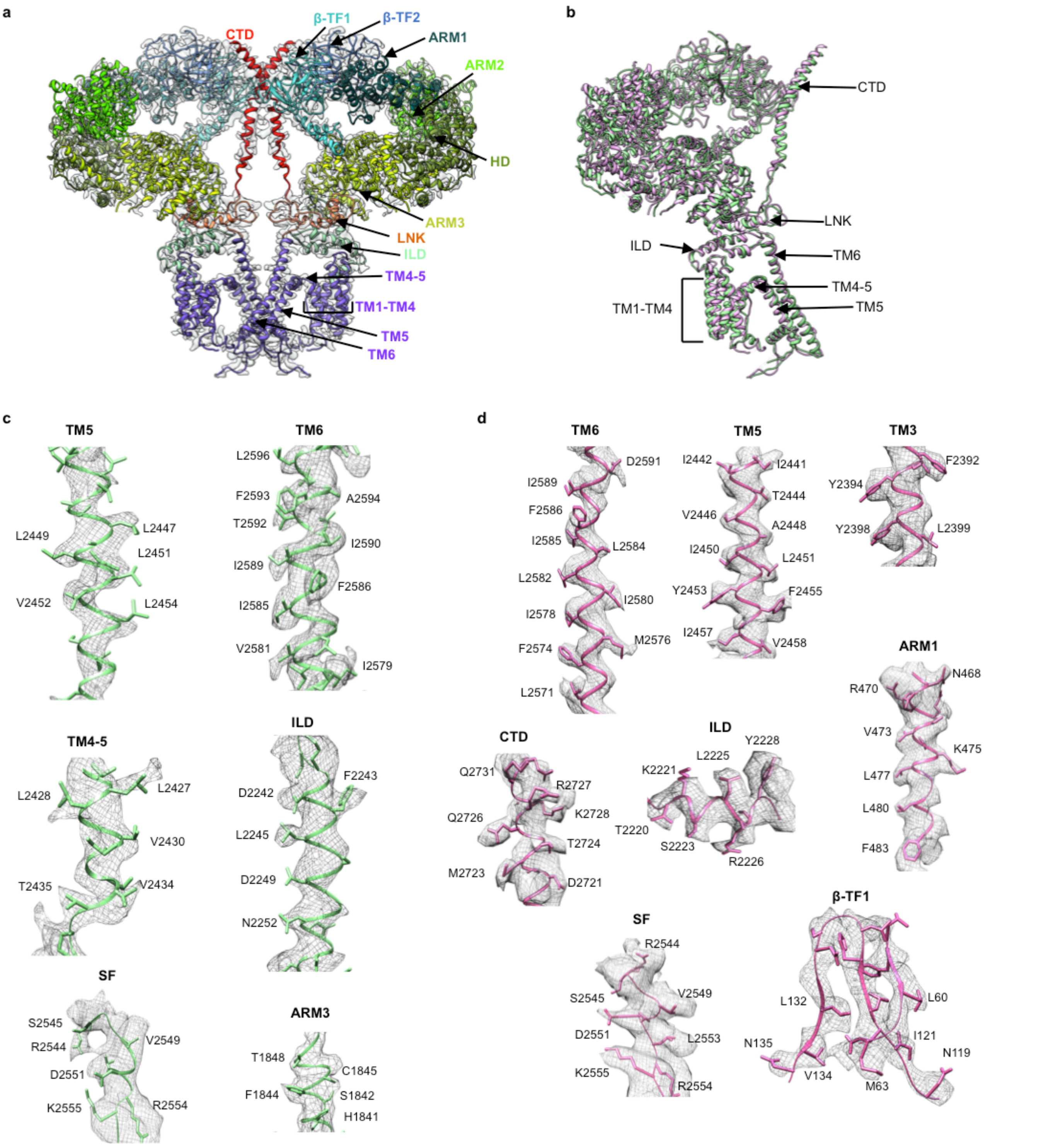

Supplementary information, Figure S5. Representative cryo-EM densities. a, The cryo-EM density map for AdA-$InsP_3R1$ is overlaid with the model; shown are two opposing subunits. The domains are colour-coded and labeled according to Supplementary information, Table S2. **b,** Models for one subunit of Apo- (light purple) and AdA-$InsP_3R1$ (green) are overlaid. **c-d**, Representative cryo-EM densities for selected regions are overlaid with corresponding models for AdA- (**c**) and Apo-$InsP_3R1$ (**d**).
