# Supplementary material for: Cryo-EM reveals ligand induced allostery underlying InsP3R channel gating
Source: Cell Res. 2018 Nov 23;28(12):1158–70. doi: 10.1038/s41422-018-0108-5 (PMC6274648; doi:10.1038/s41422-018-0108-5)
Supplement: Supplementary file 6 — Supplementary Figure S6 [file 41422_2018_108_MOESM6_ESM.pdf]

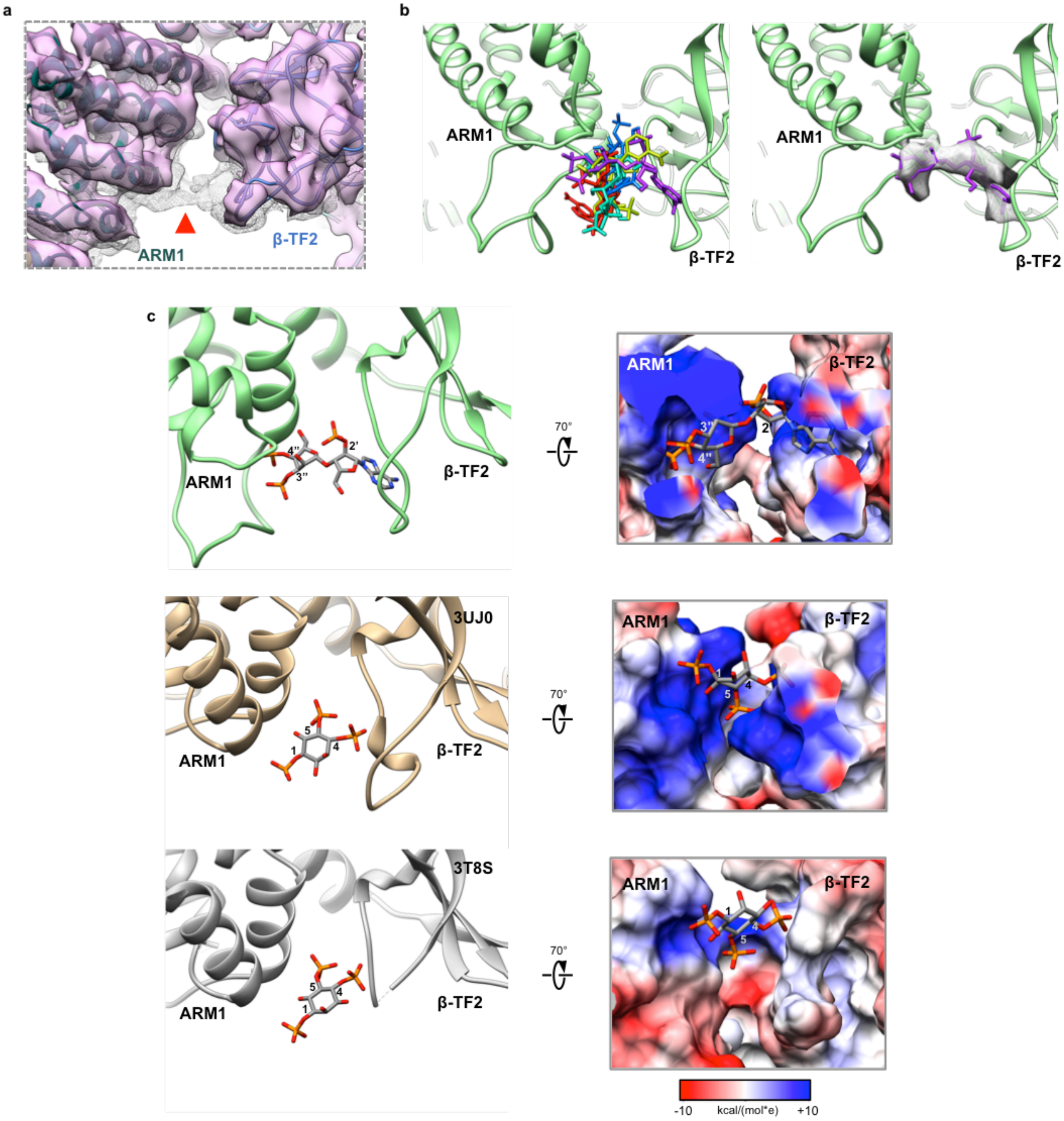

Supplementary information, Figure S6. Identification and characterization of the AdA binding pocket. a, Cryo-EM density maps for the ligand-binding pocket in the Apo- (purple) and AdA- (grey mesh) are overlaid; Apo-$InsP_3R1$ is depicted with ribbon model coloured by domain. The bridging density visualized between the ARM1 and β-TF2 domains in AdA-$InsP_3R1$ map is marked with red arrowhead. **b,** Several candidate positions for docking the AdA molecule generated using AutoDock Vina[71] (left panel) are displayed within the AdA-$InsP_3R1$ LBD structure (green). The right panel shows the final AdA molecular docking and its fit to the difference map density. **c**, Structures of isolated $InsP_3$-bound LBDs compared with the AdA-bound $InsP_3R1$ cryo-EM structure. Top panels: AdA-$InsP_3R1$ (green ribbon), middle panels: 3UJ0 (tan ribbon); bottom panels: 3T8S (grey ribbon). $InsP_3$ and AdA are colour-coded by element: (phosphorous - orange; oxygen - red; nitrogen - blue; carbon - grey; phosphates are labeled as indicated in Supplementary information Figure S1a). The right panels show the surface electrostatic charges calculated for the corresponding ligand binding pockets.
