# Supplementary material for: Cryo-EM reveals ligand induced allostery underlying InsP3R channel gating
Source: Cell Res. 2018 Nov 23;28(12):1158–70. doi: 10.1038/s41422-018-0108-5 (PMC6274648; doi:10.1038/s41422-018-0108-5)
Supplement: Supplementary file 7 — Supplementary Figure S7 [file 41422_2018_108_MOESM7_ESM.pdf]

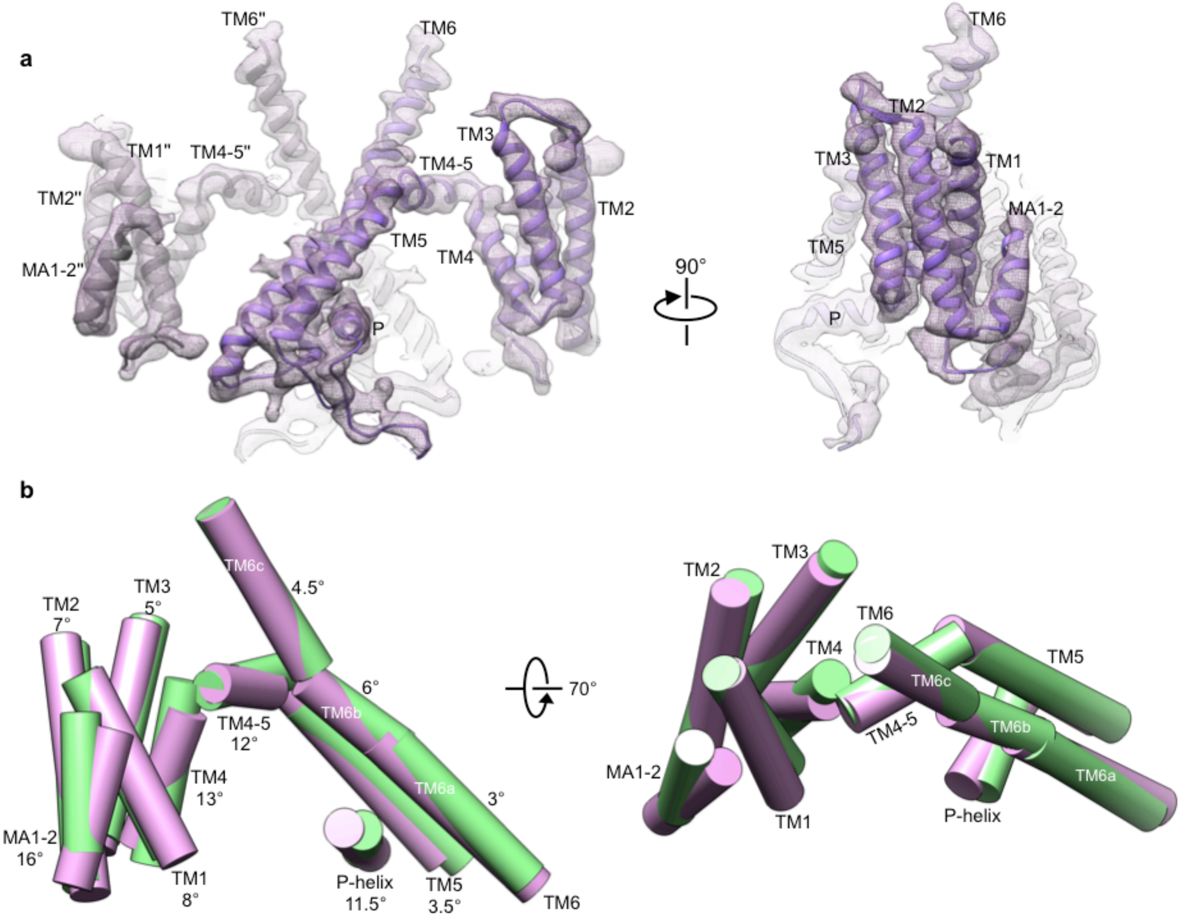

Supplementary information, Figure S7. Cryo-EM density map for the TM region of Apo-InsP$_3$R1. a, Two views of the Apo-InsP$_3$R1 cryo-EM density map for the TM1-TM6 helices and interconnecting loops of two opposing subunits; viewed parallel to the membrane plane with the lumenal side facing down. **b**, Superimposition of TM helices from one subunit of Apo (light purple) and AdA-bound (green) structures; the helices are depicted as cylinders in two views: parallel to the membrane plane (left, cytosolic side up), and rotated 70° (right, viewed from the cytosol). Changes in a rigid body tilt for each TM helix in AdA-InsP$_3$R1 with respect to the orientation in the Apo-state are indicated. TM6 helix exhibits three different tilt angles defined for TM6a (E2560-I2578), TM6b (I2579-I2590), TM6c (D2691-K2608).
