# Supplementary material for: Cryo-EM reveals ligand induced allostery underlying InsP3R channel gating
Source: Cell Res. 2018 Nov 23;28(12):1158–70. doi: 10.1038/s41422-018-0108-5 (PMC6274648; doi:10.1038/s41422-018-0108-5)
Supplement: Supplementary file 8 — Supplementary Figure S8 [file 41422_2018_108_MOESM8_ESM.pdf]

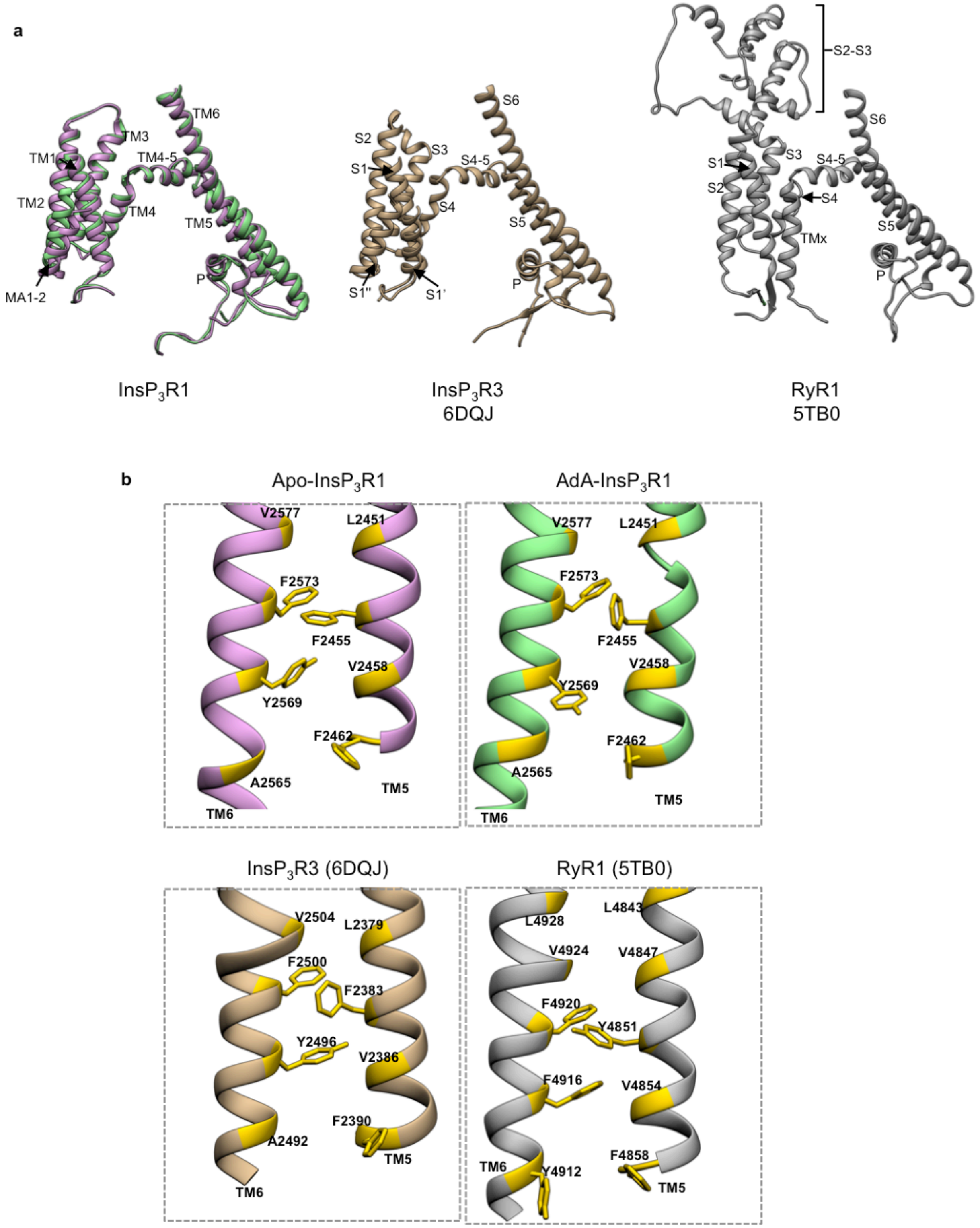

Supplementary information, Figure S8. Comparative analysis of the TM domains. a, Six TM domain structure in $InsP_3R1$ (apo-structure – light purple, AdA-structure – green), $InsP_3R3$ (6DQJ) and RyR1 (5TB0). **b,** TM6 and TM5 helices from the same subunit exhibit hydrophobic interactions and aromatic side-chain stacking in the lumenal leaflet: Apo-$InsP_3R1$ (light purple), AdA- $InsP_3R1$ (green); $InsP_3R3$ (tan), RyR1 (grey). Hydrophobic residues within the interface are colored yellow and aromatic side-chains are shown.
