# Supplementary material for: Cryo-EM reveals ligand induced allostery underlying InsP3R channel gating
Source: Cell Res. 2018 Nov 23;28(12):1158–70. doi: 10.1038/s41422-018-0108-5 (PMC6274648; doi:10.1038/s41422-018-0108-5)
Supplement: Supplementary file 9 — Supplementary Figure S9 [file 41422_2018_108_MOESM9_ESM.pdf]

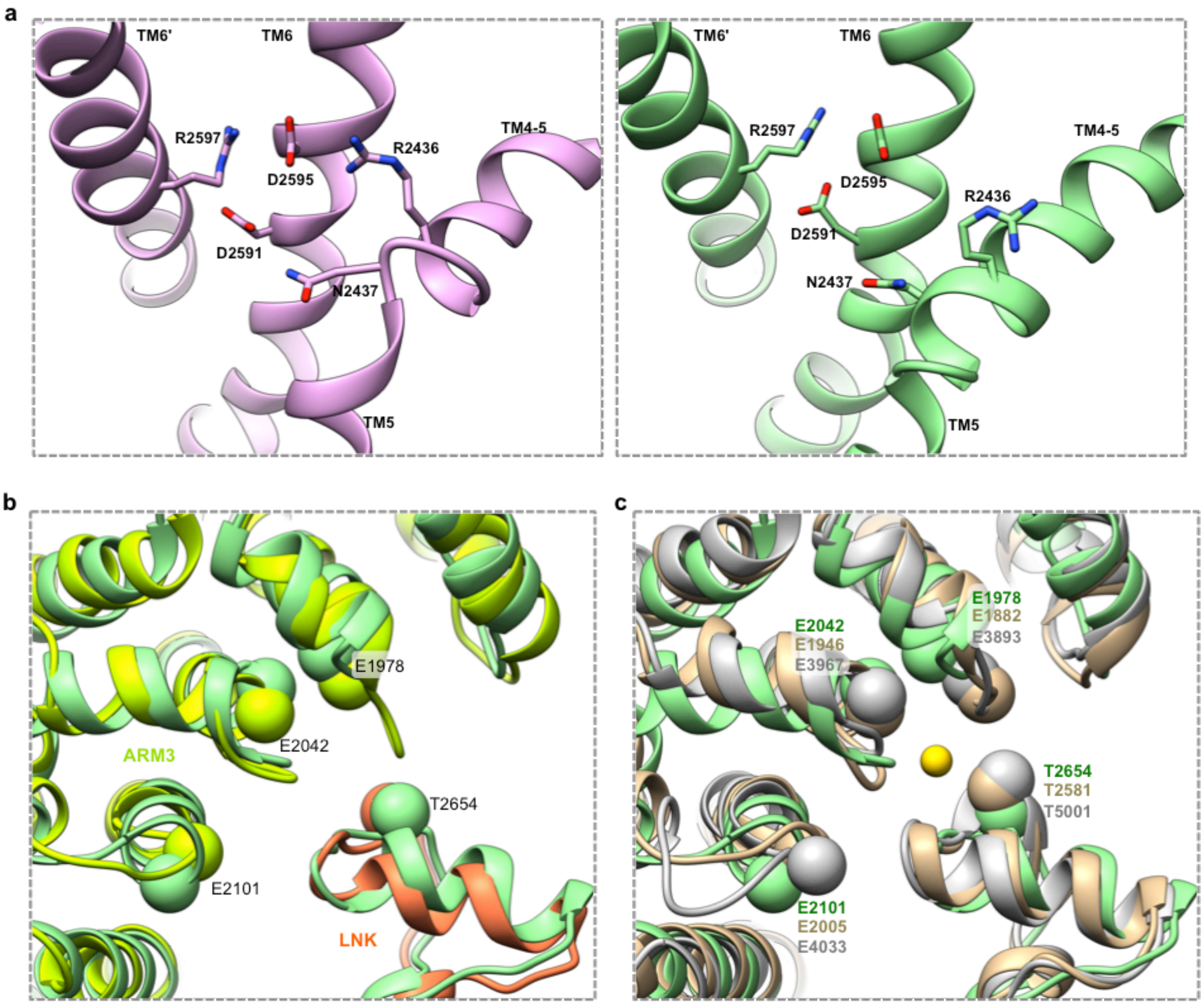

Supplementary information, Figure S9. Inter- and intra-subunit contacts within the pore region. a, Residues on the neighboring TM6 helix within 5 Å of the R2597 of TM6' are shown with side-chains. The lateral membrane-associated TM4-5 is located in close proximity to the TM6-TM6' interaction site, whereby each TM4-5 helix is positioned to interact with the TM6 helix from the same subunit. Apo and AdA-bound $InsP_3R1$ structures are depicted as light purple (left) and green (right), respectively. Conformational changes observed within this region upon ligand binding may serve to communicate with the gate. **b**, Zoomed-in view of the putative Ca^{2+} sensor region in ARM3-LNK domains of AdA-$InsP_3R1$ (green) that is superimposed with the same domain in the Apo-$InsP_3R1$ (colour-coded by domains). Residues that may play a role in Ca^{2+} binding based on a structure-based sequence alignment with RyR1[6] are labeled and their Cα atoms depicted as spheres. **c,** Structural alignment of the putative Ca^{2+} sensor regions for AdA-$InsP_3R1$ (green), RyR1 (5T15, grey) and $InsP_3R3$ (6DR2, tan). Residues involved in Ca^{2+} coordination in RyR1 and $InsP_3R3$ and the corresponding conserved residues in $InsP_3R1$ are indicated as spheres and labeled in the same color as the model. The Ca^{2+} ion modeled in RyR1 and $InsP_3R3$ structures is depicted as yellow sphere.
