# Supplementary material for: Cryo-EM reveals ligand induced allostery underlying InsP3R channel gating
Source: Cell Res. 2018 Nov 23;28(12):1158–70. doi: 10.1038/s41422-018-0108-5 (PMC6274648; doi:10.1038/s41422-018-0108-5)
Supplement: Supplementary file 10 — Supplementary Figure S10 [file 41422_2018_108_MOESM10_ESM.pdf]

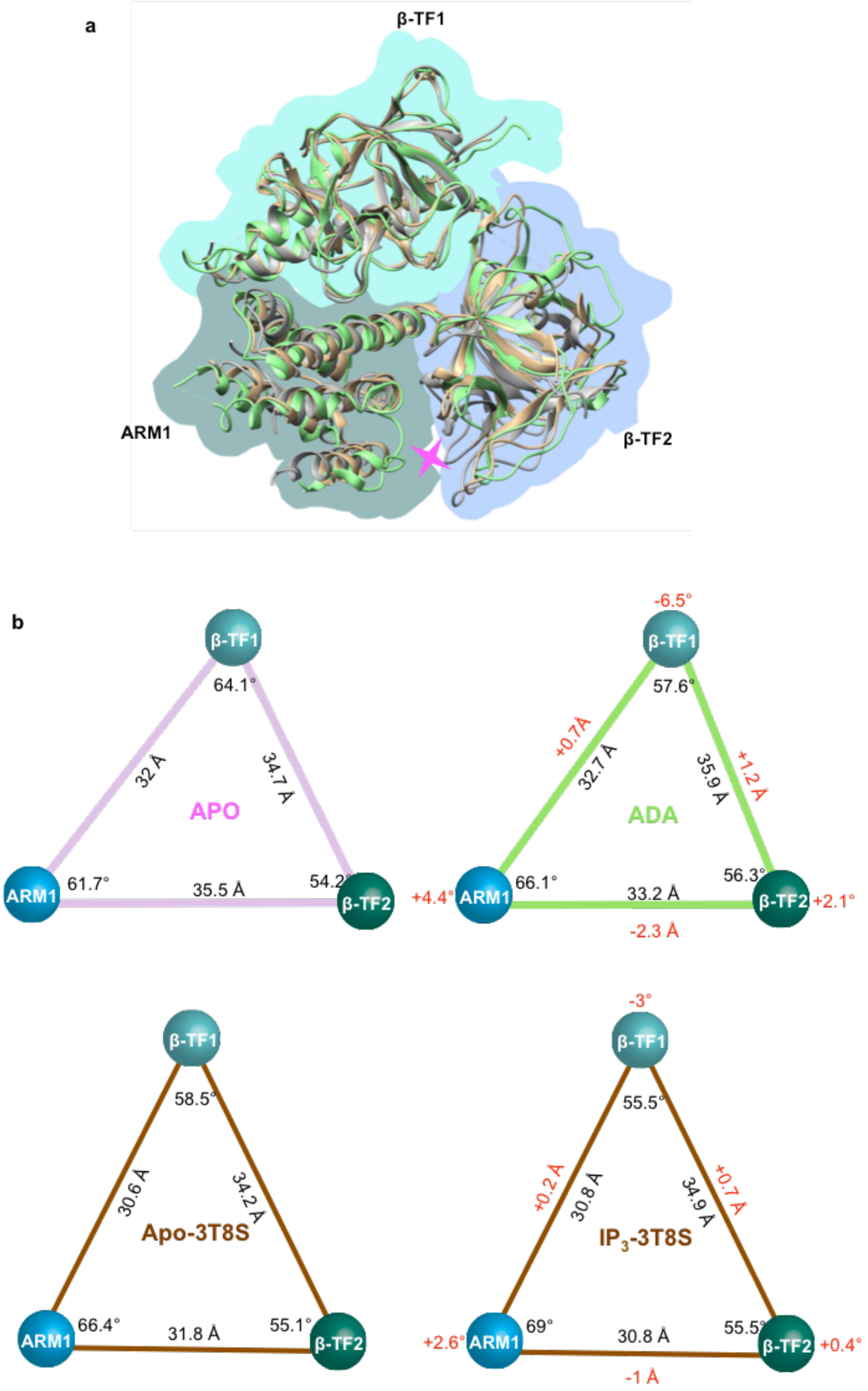

Supplementary information, Figure S10. Comparative analysis of AdA- and InsP$_3$- bound ligand binding domains. a, The triangular arrangement of ligand-binding domains within tetrameric InsP$_3$R structure: AdA-InsP$_3$R LBD (green), 3UJ0 (grey) and 3T8S (tan). **b,** Angular relationships and relative distances between β-TF1, ARM1 and β-TF2 domains in Apo- and AdA-bound structures of InsP$_3$R1 (upper panels) and in the Apo- and InsP$_3$-bound crystal structure of isolated LBD (3T8S; lower panels) as estimated based on the center of mass for each domain. It is noticeable, that AdA ligand binding results in a greater domain closure between ARM1 and β-TF2 than InsP$_3$. This results in a decrease in the angular relationship of ARM1/β-TF1/β-TF2 domains while the β-TF1/β-TF2/ARM1 angle increases.
