# Supplementary material for: Cryo-EM reveals ligand induced allostery underlying InsP3R channel gating
Source: Cell Res. 2018 Nov 23;28(12):1158–70. doi: 10.1038/s41422-018-0108-5 (PMC6274648; doi:10.1038/s41422-018-0108-5)
Supplement: Supplementary file 11 — Supplementary Table S1 [file 41422_2018_108_MOESM11_ESM.pdf]

Supplementary Information, Table S1.
Cryo-EM structure determination and model statistics

Data collection	Apo	Ligand-bound
Microscope	FEI Polara	FEI Polara
Voltage (kV)	300	300
Detector	Gatan K2 Summit	Gatan K2 Summit
Magnification	31,000	31,000
Pixel size (Å)	1.26 (0.63)	1.26 (0.63)
Total dose (e/Å^2)	38	44
Dose rate (electrons/pixel/sec)	~10	~10
Exposure time (sec)	6	7
Defocus range (μm)	-0.8 to -3.5	-0.9 to -3.2
Movie stacks	9,823	14,686
Subframes	30	35
**Data Processing**	**Apo**	**Ligand-bound**
Defocus determining software	CTFFIND3	CTFFIND3
Motion correction software	dosefgpu_driftcorr	dosefgpu_driftcorr
Refine software	RELION 1.4	RELION 1.4
Particle picking software	e2boxer.py	e2boxer.py
Number of boxed particles	207,914	191,646
Number of particles after 2D classification	144,194	179,760
Number of particles in final reconstruction	65,438	179,760
Symmetry imposed	C4	C4
Map resolution (Å)	3.9	4.5
**EMAN Refinement**	**Apo**	**Ligand-bound**
Number of particles:		
initial refinement	144,194	179,760
final reconstruction	100,615	38,405
Symmetry imposed	C4	C4
Map resolution (Å)	4.3	4.2
**3D Refinement**		**Class 4**
Refine software		RELION 1.4
Number of particles in final reconstruction		50,903
Symmetry imposed		C1
Map resolution (Å)		6.4
Symmetry imposed		C4
Map resolution (Å)		4.1
**Atomic Model**	**Apo**	**Ligand-bound**
Ramachandran outliers	0.34%	1.13%
Ramachandran favored	84.89%	84.89%
Rotamer outliers	1.49%	3.45%
C-beta deviations	7	32
RMS(bonds)	0.01	0.007
RMS(angles)	1.62	1.64
Molprobity score	2.36	3.10
Molprobity clashscore	11.27	35.36

Supplementary information, Table S1. Summary of Cryo-EM data collection, image processing, 3D reconstruction and model statistics.
