# Supplementary material for: Cryo-EM reveals ligand induced allostery underlying InsP3R channel gating
Source: Cell Res. 2018 Nov 23;28(12):1158–70. doi: 10.1038/s41422-018-0108-5 (PMC6274648; doi:10.1038/s41422-018-0108-5)
Supplement: Supplementary file 12 — Supplementary Table S2 [file 41422_2018_108_MOESM12_ESM.pdf]

## Supplementary Information, Table S2.

| Domain abbreviation | Domain Name | Spanning Residues |
|---|---|---|
| β-TF1 | beta trefoil domain 1 | M1-K225 |
| β-TF2 | beta trefoil domain 2 | W226-V435 |
| ARM1 | armadillo repeat 1 | S436-L665 |
| HD | helical domain | I707-S1008 |
| ARM2 | armadillo repeat 2 | P1025-R1538 |
| ARM3 | armadillo repeat 3 | R1598-H2192 |
| ILD | intervening lateral domain | T2193-V2265 |
| TMD | transmembrane domains | L2266-K2608 |
| LNK | linker domain | T2609-M2681 |
| CTD | C-terminal domain | S2682-A2750 |

**Supplementary Table S2.** Definition of domains in $InsP_3R1$ structure.
