# Supplementary material for: Cryo-EM reveals ligand induced allostery underlying InsP3R channel gating
Source: Cell Res. 2018 Nov 23;28(12):1158–70. doi: 10.1038/s41422-018-0108-5 (PMC6274648; doi:10.1038/s41422-018-0108-5)
Supplement: Supplementary file 13 — Supplementary Table S3 [file 41422_2018_108_MOESM13_ESM.pdf]

Supplementary Information, Table S3.

	This study	Hite *et al.*, 2018	Lin *et al.*, 2011	Seo *et al.*, 2012
**Method**	Cryo-EM	Cryo-EM	X-ray crystallography	X-ray crystallography
**Channel**	Rat $InsP_3R1$	Human $InsP_3R3$	Rat $InsP_3R1$	Rat $InsP_3R1$
**PDB**		6DQN	3T8S	3UJ0
**Ligand**	AdA	$InsP_3$	$InsP_3$	$InsP_3$
**β-TF2**<br>Loop	**Adenine**<br>T267-T276<br><br>**2' PO_3^{2-}**<br>R265	**4-PO_3^{2-}**<br>R266<br>T268<br>R270	**4-PO_3^{2-}**<br>R265<br>T267	**4-PO_3^{2-}**<br>R265<br>T267<br>R269
**ARM1**<br>Helix	**4'' PO_3^{2-}**<br>R504<br>Q507<br>K508<br><br>**2' PO_3^{2-}**<br>R511<br>K508	**5 PO_3^{2-}**<br>K507<br>R510<br><br>**1 PO_3^{2-}**<br>R503	**5 PO_3^{2-}**<br>R511	**5 PO_3^{2-}**<br>R504<br>R511<br><br>**1 PO_3^{2-}**<br>R504
**ARM1**<br>Loop	**3'' PO_3^{2-}**<br>D566<br>Y567<br>R568<br>K569<br><br>**4'' PO_3^{2-}**<br>Y567<br>N570<br>Q571	**1 PO_3^{2-}**<br>R568<br><br>**5 PO_3^{2-}**<br>Y567<br>K569	**1 PO_3^{2-}**<br>R568<br><br>**5 PO_3^{2-}**<br>Y567	**1 PO_3^{2-}**<br>R568<br><br>**5 PO_3^{2-}**<br>Y567<br>K569

Supplementary Table S3. Comparative analysis of $InsP_3$ and AdA interactions with LBD in X-ray crystallographic structures[18,19] and in cryo-EM structures of $InsP_3R1$ and $InsP_3R3$[20]. The table includes residues located within 5 Å from AdA molecule in the AdA-$InsP_3R1$ structure and may potentially coordinate the ligand in the binding pocket. Residues for coordination of $InsP_3$ were extracted from the publications indicated in the table.
