# Supplementary material for: Cryo-EM reveals ligand induced allostery underlying InsP3R channel gating
Source: Cell Res. 2018 Nov 23;28(12):1158–70. doi: 10.1038/s41422-018-0108-5 (PMC6274648; doi:10.1038/s41422-018-0108-5)
Supplement: Supplementary file 17 — . [file 41422_2018_108_MOESM17_ESM.docx]

**Supplementary Movies Legends**

**Movie S1. Structural rearrangements of cytosolic domains in InsP_3_R1 upon ligand-binding**. Movie shows a morph between Apo- and AdA-InsP_3_R1 models; the LBDs and CTDs are first coloured by subunit, then colour-coded by domain, followed by a zoomed-in view of one LBD and CTD.

**Movie S2. Ligand-evoked global conformational changes in tetrameric InsP_3_R1.** This animation shows a morph between Apo- and AdA-bound models for one InsP_3_R1 protomer colour-coded by domain, while the other subunits are statically depicted in the Apo-state and coloured white. The channel is shown in a side view parallel to the membrane plane.

**Movie S3. Structural rearrangements at the interface between the CY and TM regions.** This animation shows the morph between tetrameric Apo- and AdA-InsP_3_R1 models at the CY/TM interface; the model is colour-coded by domain and viewed along the four-fold axis from the cytosol.
